# Sequence and structural determinants of efficacious de novo chimaeric antigen receptors

**DOI:** 10.1038/s41551-026-01790-9

**Published:** 2026-09-09

**Authors:** Arthur Chow, Hoyin Chu, Ruofan Li, Benan N. Nalbant, Abdul Vehab Dozic, Laura C. Kida, Zeyu Tang, Joseph R. Palmeri, Caleb A. Lareau

**Affiliations:** 1 Computational and Systems Biology Program, Memorial Sloan Kettering Cancer Center, New York, NY, USA.; 2 These authors contributed equally: Arthur Chow, Hoyin Chu, Ruofan Li.

## Abstract

Advances in generative protein design using artificial intelligence (AI) have enabled the rapid development of binders against heterogeneous targets, including tumour-associated antigens. Despite extensive biochemical characterization, these novel protein binders have had limited evaluation in candidate therapeutics, including chimaeric antigen receptor (CAR) T cells. Here we synthesize generative protein design workflows to screen 1,758 newly designed protein binders targeting BCMA, CD19 and CD22 for efficacy in scalable protein-binding, T-cell activation and in vivo killing assays. We characterize three main challenges that hinder the utility of de novo protein binders as CARs, including tonic signalling, occluded epitope engagement and off-target activity. We develop computational and experimental heuristics to overcome these limitations, including screens of sequence variants of individual parental structures, that retain on-target CAR activation while mitigating liabilities. Together, our framework accelerates the development of AI-designed proteins for future preclinical therapeutic screening, helping enable a new generation of cellular therapies.

Chimaeric antigen receptors (CARs) are synthetic proteins that redirect the specificity of immune cells towards a restricted set of antigens^1^, enabling the precision targeting of pathogenic cells that underlie cancer^2^ and autoimmunity^3^. Autologous CAR T cells have emerged as a transformative treatment modality for haematological malignancies, with seven FDA-approved therapies demonstrating robust clinical efficacy and durable patient responses^2–4^. Effective tumour killing requires synthetic binders that sensitively and specifically recognize tumour-associated antigens (TAAs), which are incorporated into CAR transgenes with T-cell activation domains^2^. Although most clinical-stage CARs have predominantly repurposed single-chain variable fragments (scFvs) for antigen recognition, in recent years, alternative protein binder formats have demonstrated promising preclinical efficacy by engaging TAAs and controlling malignant cell growth^5^.

Artificial intelligence (AI)-driven protein binder design is an emerging method for engaging TAAs, where novel protein binders are designed against target antigens de novo^6^. AI-designed proteins offer several theoretical advantages over traditional antibody-derived domains, including rapid generation in silico^7–9^, enhanced stability through optimized folding^10–12^, small size (~60 amino acids) and prospective identification of the binding epitope^7,8,13^. These attributes of de novo protein binders stand to overcome efficacy limitations reported from various scFv-based CARs, including tonic signalling due to binder aggregation^14^ and enhanced immune synapse formation from rational epitope selection^15^. Motivated by recent work confirming the feasibility of AI-designed binders as CARs against surface molecules^16–19^, we sought a comprehensive profiling of de novo designed proteins as efficacious antigen engagers, including an elucidation of the properties that elicit effective or ineffective CAR activation and signalling.

Here we evaluate the feasibility, structural correlates and sequence determinants of efficacious CAR function with variable antigen-binding proteins created via generative protein design. We synthesize protein design pipelines that generate novel de novo binders against three antigens with clinical-stage scFv sequences as gold standards for benchmarking. Using a combination of scalable protein-binding and cell-based CAR assays, we study the binding and CAR signalling attributes of 1,758 distinct AI-designed TAA-binding proteins. Through this characterization, we identify three key constraints limiting de novo designed binders for CAR T-cell efficacy, including tonic signalling, epitope occlusion from native ligands and off-target binding. Critically, we demonstrate that these limitations can be overcome through rational protein design and engineering of non-interface residues. Our work provides a roadmap for developing next-generation CAR T-cell therapeutics with enhanced efficacy and reduced liabilities, accelerating the utilization of de novo protein binders in future cell therapies against heterogeneous antigens.

## Results

### De novo design of protein binders

We first assessed the feasibility of designing protein binders de novo against B-cell maturation antigen (BCMA), a surface protein overexpressed in multiple myeloma^20^ (Fig. 1a). We integrated various computational methods for two primary de novo binder design workflows (Supplementary Note 1). First, we implemented an RFdiffusion-based pipeline^9^, which spans backbone generation via RFdiffusion^8^, sequence diversification via ProteinMPNN^21^, and in silico co-folding validation via AlphaFold2-multimer^22^. Second, we implemented BindCraft^7^, which generates protein binder sequences via protein hallucination by iteratively optimizing amino acid compositions using AlphaFold2 gradients. As part of our workflows, we applied the recommended tool-specific filters, such as interchain predicted alignment error (ipAE) and predicted local distance difference test (pLDDT), to ensure high-confidence binding between the antigen target and designed de novo binders^9,23^ (Methods). For RFdiffusion, hotspots on BCMA (PDB 1XU2) were selected manually or via PeSTo^24^; for BindCraft, default 4-stage multimer hallucination was used without specified hotspots. Binders were generated across a range of lengths per campaign and ranked by ipAE (RFdiffusion) or interface-predicted template modeling (ipTM; BindCraft) before selection (Methods and Supplementary Note 1). Once candidate molecules were generated and prioritized from these in silico metrics for both methods, synthetic DNA encoding 100–125 designed proteins (size 35–80 amino acids) was ordered from IDT and transformed as a pool into *S. cerevisiae* with a pETcon(−) yeast surface display (YSD) plasmid and individually into *E. coli* with a recombinant *E. coli* protein expression plasmid (Extended Data Fig. 1a,b). Binding was assessed in a pooled setting via flow cytometry of fluorescently labelled recombinant BCMA protein and quantitatively confirmed individually with recombinant protein via biolayer interferometry (BLI; Fig. 1a and Methods).

To identify candidate BCMA binders, we executed 6 different design campaigns to target BCMA with YSD validation (Supplementary Table 1 and Methods). For all yeast pools, we incubated yeast displaying protein de novo binders (hereafter, ‘minibinders’) with 100 nM BCMA and off-target antigens as control to verify the specificity of BCMA binding (Fig. 1b and Extended Data Fig. 1c,d). Across our 6 campaigns, we observed the largest enrichment with the singular BindCraft campaign relative to any of the 5 RFdiffusion campaigns, as 10.7% of induced yeast bound BCMA at 100 nM, compared to a maximum of 2.4% from the RFdiffusion campaigns (Fig. 1c). Magnetic-activated cell sorting (MACS) with recombinant BCMA protein was used to enrich yeast cells that bound the target antigen at variable concentrations before PCR-amplifying and sequencing the binder-encoding inserts (Supplementary Note 2). From our BindCraft campaign, we identified 17 sequences that showed compelling increase in abundance from MACS enrichment and sequencing (Fig. 1d, Extended Data Fig. 1e and Supplementary Table 2). To confirm binding, 6 candidate sequences with high abundance in the enriched pool and high fold-changes relative to the parental pool were recombinantly expressed in *E. coli* and purified. BLI then confirmed BCMA binding, with affinities ranging from ~2–200 nM (Fig. 1e and Extended Data Fig. 1f). While RFdiffusion produced a lower overall success rate of experimental binding, parallel workflows of YSD enrichment, sequencing and individual sequence characterization via BLI confirmed BCMA binding for 2 candidate sequences from the RFdiffusion 5 campaign (Extended Data Fig. 1g,h). Together, both RFdiffusion and BindCraft feasibly generated zero-shot de novo binders with affinities comparable to those used in established therapies, and BindCraft in particular offered a novice-friendly workflow, consistent with reports from public binder design competitions^7,23^.

### Characterization of CAR activities

Following the characterization of our de novo anti-BCMA binders, we sought to evaluate their performance as CARs using both Jurkats as an in vitro cell model and primary human T cells for ex vivo assessment (Fig. 2a and Extended Data Fig. 2a). We used a second-generation CAR backbone with a 4–1BB co-stimulatory domain and fluorescent reporter protein (GFP) that enabled modular integration of new binders into the CAR backbone for testing (Extended Data Fig. 2b and Methods). We assessed tonic, off- and on-target signalling by co-culturing CAR T and Jurkat cells with various tumour cell lines expressing variable, if any, levels of BCMA, including engineered lines (Fig. 2a, Extended Data Fig. 2c and Supplementary Note 3). We used CD69 surface expression on CAR^+^ cells as a primary readout, as CD69 is rapidly expressed following T-cell activation, including CAR engagement^25,26^. We quantified the percentage of CD69^+^ cells among CAR^+^ cells, which enables a rapid and scalable assay for measuring CAR activity in Jurkats^26^. We compared the efficacy of our de novo binders to the C11D5.3 scFv, the anti-BCMA binder used in the US Food and Drug Administration (FDA)-approved CAR Abecma^27^.

First, to verify antigen binding, we confirmed surface expression of all CARs by staining for the Myc tag (purple) and validated antigen binding by adding variable concentrations of biotinylated recombinant BCMA protein (blue) to the CAR Jurkats, which collectively verified that our binders were well folded in mammalian cells (Fig. 2b). After 16 hours of co-culture, we characterized the 17 binders from our BindCraft1 campaign, observing that 9 had low CAR CD69 activation (L1–L9; 2–43% CD69^+^ CAR^+^ cells), 3 featured high tonic signalling (T1–T3; 46–70%), and 5 had low tonic but high BCMA-dependent activation (B1–B5; 22%–90%), which was qualitatively similar to the gold-standard C11D5.3 scFv (72–85%; Fig. 2b). Notably, none of the binders showed meaningful evidence of off-target activity as determined by limited CD69 activation of BCMA^−^ lines or against the CAR Jurkats alone. Structurally, each designed binder was predicted to fold into α-helical monomers with high-confidence in silico quality metrics but variable arrangements in complex with BCMA (Fig. 2c). For ease of interpretation, we defined activity gain as the difference in CD69 positivity between antigen-positive co-cultures and CAR-modified cells alone. This metric showed strong correlations with multiple in silico design metrics, most notably average differentiable solvent accessible surface area (dSASA; Spearman *ρ* = 0.72, *P* = 0.0017; Fig. 2d), with the top-performing binders B2, B4 and B5 showing the highest overall BCMA-dependent CD69 activity gain (Fig. 2e). While our BindCraft-derived de novo binders exhibited clear antigen-specific activation, we also confirmed CAR activity from our two RFdiffusion-derived binders in analogous CAR Jurkat activation, consistent with recent reports^17,19^, albeit to a lesser degree than our binders from BindCraft (Extended Data Fig. 2d,e).

Encouraged by these results, we screened the best BindCraft binders (B2, B4, B5) in primary CAR T cells (Methods). We again observed strong BCMA-dependent CAR T activation, especially for the B5 binder (52–74% CD69^+^; Fig. 2f). Conversely, while our B4 binder demonstrated strong BCMA-specific activity in our Jurkat assay, it failed to recapitulate the same level of activation in primary CAR T cells (Extended Data Fig. 2f). Thus, we assessed our two best de novo binders (B2 and B5) for hallmarks of productive CAR signalling response, including production of effector molecules and cytokines IL-2, IFNγ, TNF and Granzyme B following co-culture with the same cell lines via enzyme-linked immunosorbent assays (ELISAs) in three distinct T-cell donors (Methods). For all four molecules, binding of the B5 minibinder induced comparable or higher levels of production of effector molecules compared with the clinical-stage BCMA scFv (Fig. 2g,h). We verified consistency of key co-culture readouts comparing B5 and the C11D5.3 scFv in triplicate (Extended Data Fig. 2g). Together, these data confirm the feasibility of de novo minibinders as CAR antigen receptors, consistent with other reports of de novo binders targeting human and viral antigens^16–19^.

### Sequence variation underlies tonic signalling

In general, de novo designed minibinders that pass our chosen designability metrics fold into well-defined structures as predicted by state-of-the-art folding algorithms with high in silico confidence^9^. Unlike scFvs that engage antigens from complementarity determining region (CDR) loops, de novo binders fold into rigid, stable structures that typically bind target antigens along non-polar residue sites. From these high-confidence backbones, algorithms such as ProteinMPNN can generate hundreds of high-quality candidate sequences for an individual structure^21,28,29^. Hence, we reasoned that our design pipeline could sample sequence variation from a selected binder (that is, B5), enabling correlative analyses to identify sequence and biophysical parameters that underlie efficacious CAR function.

To ensure that the mutagenized binder sequences have high sequence variation while preserving high likelihood of BCMA binding and structural similarity, we developed the CARPNN (chimaeric antigen receptor-protein-mpNN) workflow which diversifies the binder sequence at interface and non-interface residues and filters candidates on the basis of BindCraft-inspired, Boltz-2 (ref. 30)-based filters. In particular, starting with our parental B5 binder, we applied ProteinMPNN trained with only soluble structures (SolubleMPNN)^31^ to generate a total of 4,000 sequences that mutagenized residues critical to the CAR synapse (<4 Å) or that were not part of the binding interface (Fig. 3a and Methods). These candidate sequences were then refolded against BCMA via Boltz-2 (ref. 30) and the average metrics across 5 diffusion samples such as pLDDT and dSASA were used for design filtering (Methods). Among the filtered sequences, we selected 21 additional CAR sequences that diversified over the number of interface hydrogen bonds, hydrophobic residues, charge and dSASA with a minimum amino acid Levenshtein distance of at least 6 and maximum backbone root mean square deviation (RMSD) of 1 Å compared to the B5 parental binder (Fig. 3b and Supplementary Table 2). Overall, our selected binders had a median of 9 residues changed at the interface and a median of 21 changed from non-interface residues, with a total of 81% of possible residues mutated at least once (Extended Data Fig. 3a). Among our selected binders, each had similar, if not improved, in silico performance metrics as the parental B5 binder, including complex interaction metrics correlated with performance of de novo binders^9^ (Extended Data Fig. 3b).

Synthetic DNA encoding each of these 21 binders was purchased from IDT and cloned into the same CAR construct, and CAR Jurkats were screened in parallel (Fig. 3c). We observed binding of recombinant BCMA from all diversified sequences, with modest variation at lower antigen concentrations. Co-culture of CAR Jurkats with BCMA-positive or negative cell lines showed considerable variance in CAR activation across conditions, including the B5.I0 sequence with improved activity relative to the B5 parental binder and comparable activity to the C11D5.3 scFv used in Abecma (Fig. 3c and Extended Data Fig. 3c).

We observed 5 sequences with substantial tonic signalling and an overall activation gradient in this experiment ranging from ~0 to ~80% CD69 expression in the CAR-alone condition. As all proteins had similar predicted folds, confidence metrics and BCMA binding, we attributed the variation in tonic signalling to underlying sequence properties of the BCMA binders. To assess the impact of the diversified biophysical and sequence metrics, we performed correlations of the tonic signalling and sequence attributes of these 22 CARs, observing a strong association between the binder net charge and the degree of tonic signalling (*r* = 0.65; *P* = 0.00099; Fig. 3d). Of note, no other biophysical parameter showed a strong correlation with CAR tonic signalling or activity gain, including hydrophobic content, number of hydrogen bonds, or dSASA (Extended Data Fig. 3d). These analyses implicate binder net charge as a determinant of tonic signalling in de novo designed CAR T cells and a promising filtering heuristic for ensuring efficacious CARs. Our findings corroborate the report of positively charged patches (PCP) on scFvs as a major determinant of tonic signalling and a heuristic to computationally optimize for improved CAR function^32,33^.

We next sought a more comprehensive characterization of our lead de novo binders that showed comparable T-cell activation. To assess this, we first performed single-cell RNA sequencing (scRNA-seq) on primary CAR T cells expressing C11D5.3, the parental B5 binder and three evolved minibinders (B5.I0, B5.N6, B5.N9) following co-culture with K562 cells overexpressing BCMA under identical conditions for all constructs (Supplementary Note 4). The transcriptional profiles of the CAR T cells recapitulate the activation status seen in Jurkat screening, where cells expressing binders with strong antigen-dependent activation (C11D5.3, B5, B5.I0) clustered separately from those with minimal activity gain (B5.N6, B5.N9; Fig. 3e,f and Extended Data Fig. 3e). Module scoring of CAR T cells revealed strong effector activation accompanied by low exhaustion signatures for the C11D5.3, B5 and B5.I0 constructs, consistent with their high CD69 responses on BCMA^+^ target cells and low activation on BCMA^−^ cells (Extended Data Fig. 3f). As expected, the cluster containing C11D5.3, B5 and B5.I0 overexpressed effector cytokines such as *IFNG* and *TNF* (Fig. 3f). In addition, cells with evolved binder B5.I0 had significantly higher expression of activation markers such as *IL2* and *IFNG* compared with the parental binder B5 (Fig. 3g, Extended Data Fig. 3g and Supplementary Table 3). Stratifying into CD4^+^ and CD8^+^ subsets showed little difference in CD4^+^ proportions between constructs and largely concordant differential expression with similar effector and exhaustion module scores across subsets (Fig. 3h, Extended Data Fig. 3h–j and Supplementary Note 4).

### Tumour control via de novo CARs

We next hypothesized that our de novo CAR T cells would enable efficacious tumour control. Across three distinct primary T-cell donors, we engineered CARs containing variable binders for evaluation in in vitro killing assays (Methods). Compared to the parental B5 CAR and the scFv-based CAR, our evolved B5.I0 CAR further demonstrated equivalent or enhanced tumour cell control across additional BCMA^+^ cell lines, including RPMI-8226, engineered K562^−^BCMA^+^ cells and Raji cells (Fig. 4a and Extended Data Fig. 4a,b).

We next assessed the efficacy of our de novo CARs in vivo. We established an RPMI-8226 xenograft model and treated mice with vehicle (PBS), untransduced T cells (UTD), the C11D5.3 clinical scFv, or our evolved B5.I0. We monitored tumour burden weekly by bioluminescence imaging over 4 weeks (Fig. 4b and Methods). CARs engineered with either the C11D5.3 or B5.I0 binder drove sustained tumour regression to baseline within 2 weeks, whereas mice treated with the vehicle or UTD T cells showed continued tumour outgrowth (Fig. 4c,d and Extended Data Fig. 4c–e). Quantification of circulating CAR T cells in peripheral blood confirmed in vivo persistence of both constructs 3.5 weeks post treatment, with the B5.I0 treated animals harbouring slightly elevated levels of CAR^+^ cells (Fig. 4e; *P* = 0.061).

We then quantified the proliferative capacity and potential effector function of these long-lived CAR T cells via an in vitro repetitive stimulation assay. Transduced T cells were repeatedly challenged with RPMI-8226 target cells, with proliferation assessed at each round and killing at the final round (Fig. 4f and Methods). The B5.I0 CAR T exhibited strong proliferative capacity during the first three rounds of antigen challenge, exceeding both C11D5.3 and the parental B5 binder (Fig. 4g). Conversely, as a control, the empty control showed minimal expansion across all stimulations. After 4 rounds of repeated stimulation at high antigen levels, a final 1:1 co-culture for all CARs converged to comparable levels, each displaying minimal cytotoxic capacity after repeated antigen challenge (Fig. 4h). These results suggest that our engineered CAR binders can alter expansion kinetics but require additional T-cell engineering beyond the second-generation CAR format to maintain cytotoxic function after repeated antigen stimulation.

Given B5.I0’s enhanced proliferative response to antigen, we hypothesized that its functional advantage could emerge under conditions where effector expansion is rate limiting. To assess this, we implemented a second in vivo model with a low E:T ratio of 1 effector cell to 10 tumour cells (Fig. 4i). Under these high tumour burden conditions, B5.I0 CAR T cells achieved near-complete tumour control, whereas either clinical-grade binder, C11D5.3 or VHH1-VHH2 (a best-in-class biparatopic binder used in cilta-cel or CARVYKTI^34^), failed to achieve tumour control at this ratio (Fig. 4j,k and Extended Data Fig. 4f–h). Together, these characterizations demonstrate that de novo designed CARs can be engineered to possess favourable properties for improved tumour control in high tumour burden preclinical models. However, our results also indicate that these CARs may lose cytotoxic potential in a second-generation CAR format, consistent with observations made with clinical CARs^1^, motivating synergistic modifications to enhance T-cell fitness beyond the binder itself.

### Epitope occlusion by native co-factors

A conceptually attractive aspect of computational binder design is the pre-specification of residues for binding on the target surface. For smaller targets such as BCMA (36-amino-acid extracellular domain), there are few potential contact residues, limiting the diversity of epitope binding. Conversely, for CD19, a B-cell-restricted surface antigen targeted by multiple FDA-approved CAR T products for B-cell leukaemia and lymphoma, a larger extracellular domain (~280aa) results in more candidate epitopes, noting that proximity to the tumour cell surface may be advantageous^35^.

To contextualize the geometry of CD19, we superimposed the crystal structure of CD19 with the widely used FMC63 scFv used in clinical CAR T cells (PDB 7URV) and a crystal structure with its natural binding partner CD81 (PDB 7JIC; Fig. 5a). We then launched six distinct binder campaigns with variable hotspot residues on CD19 to develop de novo binders (Supplementary Table 1 and Methods). To our surprise, regardless of the input parameters, nearly all binders passing in silico design filters were constrained to the same CD81 binding epitope, probably reflecting that generative methods are prone to copying known protein complex interfaces and are biased towards hydrophobic patches (Fig. 5b). YSD screening of synthesized binders again yielded an enrichment for CD19 binders developed via BindCraft (BC2, 10.0%) with no clear evidence of binding for any of the RFdiffusion campaigns (RFD1–4, 1–3%; Fig. 5c,d). The major difference between campaigns BindCraft1 and BC2 was the overall size of the binder, which we scaled from 50–65 amino acids to 100–200 amino acids (Methods). Although emerging protein design methods may enable targeting geometric matches^36^ and disordered residues^37,38^, our results reflect that the current generation of widely used binder design tools are limited in their ability to position binders onto physiologically relevant surfaces for certain target antigens.

From our BC2 campaign, we identified three enriched sequences that bound to CD19, which we subsequently expressed as CARs into Jurkat cells (Fig. 5e–g). For one CD19 binder, C3, we did not observe recombinant CD19 binding when expressed as a CAR in Jurkat despite a strong pooled enrichment following MACS (Fig. 5f), reflecting that yeast will not always translate into binding for human cells. Thus, we recommend staining CAR Jurkats with fluorescently labelled recombinant antigen to discriminate binders subject to this limitation. Otherwise, CAR Jurkats expressing the enriched binders C1 and C2 showed limited activation in co-cultures despite their clear binding to recombinant antigen (Fig. 5g).

Motivated by our success in diversifying the underlying sequences from our parental BCMA binder, we reasoned that our CARPNN workflow could produce sequence variants with improved CAR activity (Extended Data Fig. 5a–f). However, consistent with the limited CAR activity observed in our initial Jurkat co-culture screen, the CARPNN-evolved binder C1 and C2 variants did not show any measurable improvement for antigen-specific activation (Extended Data Fig. 5c,f). Notably, mutations within the binding interface ablated CD19 binding, suggesting that the CD81-associated target epitope is geometrically constrained and affords minimal tolerance for sequence variation (Extended Data Fig. 5c,f).

Despite >10 minibinder proteins binding recombinant CD19 in Jurkats, we did not observe greater than a 10% activity gain in any of the parental or evolved de novo binders (Fig. 5h). Conversely, the FMC63-expressing CARs displayed both high antigen affinity (96% binding at 10 nM CD19) and potent antigen-specific activation (90.2%, CD69 activity gain). We reasoned that this disparity could be attributed to the steric hindrance as our minibinders targeted the same epitope as the endogenous CD81, which could explain the lack of antigen-specific activation. To test this, we generated CD81-knockdown derivatives of CD19-overexpression K562 target cell lines and re-evaluated C1 and C2 minibinders in CAR co-culture (Methods). Strikingly, previously non-functional binders (C1 and C2) drove activation of CD69 in CAR Jurkats co-cultured with specifically CD81-knockdown target cells (Extended Data Fig. 5g). Further, in vitro killing assays demonstrated cytotoxicity in primary T cell co-cultures despite altered growth kinetics of the CD81-knockdown cells (Fig. 5i). Conversely, FMC63, which engages an epitope geometrically distinct from the CD81 interface, resulted in T-cell activation regardless of CD81 expression (Fig. 5i). Together, these data indicate that although designed binders can recognize recombinant CD19, the dominant CD19 epitope targeted by the de novo binders overlaps with the region occluded by CD81 in the native CD19–CD81 complex and blocks CAR recognition of the CD19 antigen (Fig. 5j). Additional campaigns targeting the FMC63 epitope using more recent binder design tools, including BoltzGen and RFdiffusion3, also failed to generate stable binders after testing an additional 67 single-domain antibodies (VHHs) and 102 minibinders (Supplementary Note 5). Together, our results suggest that additional iterations of protein design tools may be required to generate binders against biophysically unfavourable but therapeutically important residues, a critical capability given the competition for binding interfaces with endogenous ligands.

### Identifying and rescuing off-target CAR activity

Having established functional CAR binders targeting the two antigens with FDA-approved products, we sought to develop CAR-compatible minibinders against an earlier-stage target, CD22 (refs. 39,40). RFdiffusion and BindCraft campaigns yielded protein binders with strong in silico success metrics against a hydrophobic patch within domain 7 of CD22 (Fig. 6a and Supplementary Table 1). YSD and sequencing again confirmed the high experimental success rate of BindCraft (1.9%), resulting in 4 candidate binders (D1–D4) that were screened using our CAR Jurkat workflow (Fig. 6b,c). Next, recombinant antigen flow and on-target CAR activation against CD22-expressing cells confirmed on-target activation with minimal tonic signalling (Fig. 6d). Surprisingly, we also observed a gain in CD69 expression (42.9% and 59.6%) in co-cultures with the RPMI-8226 line, a verified CD22^−^ cell line, for the D1 and D2 CARs, respectively (Fig. 6d,e). Conversely, RPMI-8226-dependent CD69 activation did not occur for the CAR expressing m971 (6.2%), the clinical-stage scFv targeting CD22, which bound a distal epitope from D1 (Extended Data Fig. 6a).

To verify the putative off-target activation from the D1 binder in the RPMI-8226 co-cultures, we repeated the CAR Jurkat co-cultures with variable effector-to-tumour (E:T) ratios (Methods). The variable co-culture compositions yielded the expected results for CD22^−^ and CD22^+^ lines, where Jurkat activation progressively increased overall when in culture with greater abundance of CD22^+^ cells (Fig. 6f). For RPMI-8226, we similarly observed dose-dependent activation of the D1 de novo minibinder (up to 65%) but not the m971 scFv (max. 11%; *P* = 0.006; two-sided Wald test from linear model), confirming off-target activation from co-culture with a CD22^−^ line.

We hypothesized that our CARPNN workflow could reduce this off-target liability while retaining on-target activity. To achieve this, we generated and scored 10,000 diversified sequences based on the D1 binder, selecting an additional 23 binders for screening in CAR Jurkats for on- and off-target activation (Fig. 6g and Extended Data Fig. 6b). Remarkably, evolved sequences with sequence variation outside of the CD22 binding interface resulted in more specific CD22-dependent CAR signalling (Extended Data Fig. 6c,d). In particular, we observed one variant, D1.N0, that retained strong on-target activity, exhibiting strong recombinant binding (78% of CAR cells bound at 0.1 nM CD22) and CD22-specific activation (65–74% CD69^+^) comparable to both m971 (83% CAR cells bound at 0.1 nM CD22; 61–90% CD22-specific CD69) and the parental D1 binder. Critically, the non-interface mutagenesis greatly reduced off-target activation from co-cultures with RPMI-8226 (D1: 42% vs D1.N0: 19%), which was verified in triplicate (Fig. 6h). To verify this result, we expanded our co-culture screening across additional CD22^−^ cell lines to assess any unanticipated off-target activity. Among these, co-culture with HeLa cells again showed evidence of CD22-independent CD69 activation (48.8%) for the parental D1 binder but not the clinical scFv (7.8%) or the evolved D1.N0 variant (8.2%; Fig. 6i). Moreover, scRNA-seq profiling of primary CAR T cells encoding various minibinders confirmed on-target activation of m971, D1 and two evolved variants (D1.N0 and D1.N1), whereas the CD22 minibinders (D1, D1.N0, D1.N1) had lower scRNA-seq activation scores, probably as a reflection of their lower binding affinity to CD22 compared with the m971 scFv (Fig. 6j). In addition, activation was restricted to the D1 parental binder when in co-culture with RPMI-8226, both observations consistent with the Jurkat data (Fig. 6j and Methods). Consistent with these activation signatures, engineered D1.N0 CAR T cells in three separate donors preserved cytotoxicity and effector cytokine secretion against CD22^+^ cell lines (Fig. 6k and Extended Data Fig. 6e–g). To resolve potential off-target interactions, a computational screen intersecting the Human Protein Atlas surfaceome with GTEx expression data (Extended Data Fig. 6h and Supplementary Note 6) nominated no credible surface off-target for D1: the top candidate, CXCR4, showed a co-folding ipSAE disparity between D1 and D1.N0 but a predicted binding site that was cytosolic and inaccessible to CAR T recognition (Extended Data Fig. 6i,j).

Together, our characterization motivates the need for extensive off-target screening of de novo designed proteins and provides a facile strategy for mitigating off-target activation. As ~28% of clinical-stage binders have one or more off-target binding events^41^, we anticipate that preclinical minibinders probably bind off-target antigens at a similar or greater rate, but such events may be potentially rescued via sequence diversification and screening. As the rescuing mutations were located at non-interface residues, we emphasize that the off-target interaction could occur in non-interfacial residues, a paradigm that has been corroborated by antibody language models^42^.

### In silico attributes predict efficacious CAR designs

We hypothesized that a retrospective analysis of all campaigns could identify in silico heuristics for prioritizing sequences with a higher success rate, both for protein binding and CAR activation. First, we identified a clear increase in alanine composition among binders generated by the RFdiffusion workflow compared to BindCraft (Fig. 7a). We reason that the overrepresentation of alanine creates poorly packed hydrophobic interfaces with largely non-functional side chains, reducing stability and limiting binding despite passing other recommended in silico success metrics. Next, using Boltz-2 (ref. 30), we computed all binder sequences that we have tested in YSD and/or CAR activation assays to determine relative optimal metrics that have emerged across design tasks, including ipTM^7^, pAE interaction^9^ and ipSAE^43^. To quantify the binding affinity of binders that have been tested in Jurkats, we estimated the *K*_d_ on the basis of a nonlinear least square (NLS) fit of the staining intensity at different concentrations scaled to the known affinity of the positive controls reported in other studies^44–46^ (Methods and Extended Data Fig. 7a,b). We found that for binders tested in the CARPNN campaigns, while decrease in ipAE and increase in ipTM generally corresponded to higher-affinity binders, ipSAE was the best calibrated cut-off metric, where binders with ipSAE higher than 0.85 had the highest success rate at achieving sub-1,000 nM binding across different campaigns (Fig. 7b and Extended Data Fig. 7c). Similar behaviour was also seen in our yeast surface display campaigns, where a single ipSAE cut-off of 0.85 was consistently the best single cut-off metric across different campaigns (Fig. 7c and Extended Data Fig. 7d,e). When retrospectively applying the additional ipSAE ≥ 0.85 filter, only 10 candidates would have passed the threshold, including 30.0% (*n* = 3/10) which showed enrichment at 1,000 nM by YSD. Thus, our analyses strongly support the inclusion of ipSAE as a filtering metric for in silico prioritization in parental and ProteinMPNN-based evolution campaigns. Additional characterization of in silico metrics predicting experimental outcomes are discussed in depth in Supplementary Note 7.

We also assessed the immunogenic potential of our de novo binders relative to existing CAR scFvs^47^ using NetMHC^48^ (Methods). When normalized for length, de novo binders were no more likely to contain immunogenic peptides than clinical scFvs (Fig. 7d), and their small size yielded fewer predicted epitopes across diverse HLA alleles (Extended Data Fig. 7f). Although in vivo testing is required, we observe no evidence of increased sequence liabilities, and future campaigns could add sequence constraints to further minimize immunogenicity.

Finally, an open question is the optimal affinity window for CAR function. Clinical data from paediatric patients treated with a low-affinity CD19 CAR demonstrated that a 14 nM scFv outperformed the picomolar-affinity FMC63 scFv (~0.3 nM), suggesting that moderate affinities can yield superior T-cell expansion, safety and anti-tumour activity^49^. Using our successful binders from the BCMA and CD22 campaigns, we directly compared the staining intensity of the antigen at 1 nM to CAR activity gain (Methods). We observed a strong correlation between the staining intensity and activity gain for both BCMA (*P* = 0.00033; two-sided Spearman correlation test) and CD22 (*P* = 0.0048; two-sided Spearman correlation test), suggesting that improved affinities of de novo binders generally enhance CAR efficacy for these proteins (Fig. 7e). In contrast to scFvs that can produce picomolar-affinity binders from immunization campaigns, current tools that produce binders on the order of 1–1,000 nM probably benefit from affinity optimization. Combined with our successful application of ProteinMPNN-based redesigns once a parental structure binder has been identified, we suggest that sequence evolution, particularly at non-interface residues, can simultaneously improve affinity and CAR activity while mitigating liabilities, including immunogenic epitopes, off-target binding and tonic signalling.

## Discussion

Generative artificial intelligence has accelerated the adoption and success of de novo protein design, enabling the rapid creation of structures with potential therapeutic properties at a high success rate^6,50^. These include recent applications establishing de novo design of high-affinity binders against peptide–MHC complexes^51–53^ and surface antigens with complex topologies, including disordered regions^37^ and G protein-coupled receptors (GPCRs)^54^. In silico design is thus a compelling alternative to conventional binder discovery, enabling stand-in replacement of antibody fragments in established modalities such as drug conjugates, bispecifics and cell therapies. However, there have been limited reports guiding considerations of protein sequence design that would enable preclinical efficacy of designed proteins beyond recombinant antigen binding. Through our assessment of 1,758 binding proteins and engineering of CARPNN, our study outlines critical considerations for enhancing the efficiency of protein binder design and their use as CARs.

To generate parental protein binders de novo, we focused on the application of two leading binder design tools, RFdiffusion and BindCraft. Across our campaigns of three different target antigens, we observed a consistently higher binder design success rate in campaigns where BindCraft was used as the primary design method. This probably reflects BindCraft’s more stringent filtering, as our CARPNN campaigns achieved high success using similar Boltz-based filters. In addition, BindCraft’s performance in community-based benchmarks^23^ and its ease of use allowed for rapid comparison and facilitated fast adoption, highlighting the importance of open benchmarks and tool accessibility in protein design. In the spirit of these principles, our study presents one of the largest datasets of de novo binders measured using a standardized YSD across multiple concentrations. In addition to the data presented in our study, we also propose using ipSAE as an in silico metric for binder identification. While other studies have also identified ipSAE as an effective metric for classifying binders from meta-analysis of other published de novo datasets^55^, our results reinforce that ipSAE may be a calibrated metric across antigens to inform future design campaigns.

To enable AI-designed proteins for future candidate therapeutics, further characterization is required beyond recombinant protein binding. However, little guidance has been reported for efficacious binders in creating immune synapse for T-cell-mediated killing. Through engineering our BindCraft-derived binders against our target antigens, we identified three limitations and recommendations to overcome them (Fig. 7f). First, by diversifying the parental BCMA binder sequence, we identify that higher numbers of positive residues are associated with increased tonic signalling, consistent with a report of positively charged residues in scFvs^32^. Unlike the previous characterization that analysed 10 different target antigens^32^, our framework uniquely controls for similar structural, antigen-binding and approximate antigen affinity to assess sequence-to-function relationships of CAR activity, including tonic signalling. Second, our CD19-directed CARs bound recombinant protein but showed no activation in cell co-cultures, indicating that the designs targeted a CD19 epitope masked by CD81 in the native complex and reflecting a fundamental limitation of current generative design tools. Namely, de novo design methods may bias interfaces towards geometrically tractable surfaces rather than physiologically accessible epitopes on native multimolecular complexes. Finally, through extensive co-culture of our CD22 CARs, we characterized off-target activation from co-cultures with CD22^−^ cell lines. However, by extending our CARPNN framework, we could abrogate off-target signalling through sequence evolution of the non-interface binding residues. As co-folding analyses could not identify the putative off-target, we recommend extensive co-culture screening for off-target activation and subsequent sequence evolution to mitigate off-target activation in de novo CARs.

Although most of our characterizations of CAR efficacy occurred in simplified in vitro settings, we demonstrated that our de novo protein engineering principles translate to in vivo efficacy. Namely, our evolved BCMA-targeting binder, B5.I0, achieved tumour control comparable to or exceeding clinically approved binders in xenograft models of multiple myeloma. While these de novo designed binders can yield effective T-cell responses, our additional characterizations show that additional T-cell engineering may be required to sustain cytotoxic capabilities upon repeated antigen stimulation for sustained therapeutic efficacy. Thus, we reason that CAR constructs applicable for clinical testing may draw from both binder engineering principles that we and others^33^ report in conjunction with genetic and epigenetic engineering to T-cell circuitry to enhance cytotoxicity and diminish dysfunction^1^. Because we used a second-generation CAR backbone throughout, other factors, such as antigen density or third-/fourth-generation CAR modifications, may alter the binding kinetics required for optimal efficacy.

Looking forward, we expect that our characterization of challenges and limitations of de novo designed molecules will improve the efficiency of developing candidate molecules with improved efficacy and reduced liabilities. These efficacious binder design principles will further enable screening of more complex T-cell circuits, including multispecific formats via AND^56^, IF-THEN^57^ and NOT^58^ CAR activation. We further envision an integrated loop where comprehensive single-cell profiles of primary diseased tissues nominate optimal combinations of antigens^59^ that can, in turn, be rapidly synthesized into sensitive and specific cellular therapies enabled by generative artificial intelligence.

## Methods

### Backbone generation with RFdiffusion and BindCraft

To generate parental protein binder backbones, we employed two complementary de novo design workflows against the crystal structures of BCMA (PDB 1XU2), CD19 (PDB 7URV) and CD22 (PDB 5VKJ, 7O52), with campaign-specific parameters detailed in Supplementary Note 1. In the RFdiffusion workflow^9^, we generated binder backbones of 30–90 residues against hotspot residues selected on the basis of surface-exposed hydrophobic residues. Backbones were sequence designed with ProteinMPNN^21^ (10 sequences per backbone) and filtered using the AF2 initial-guess protocol^22^, retaining co-folds with complex pLDDT >80 and pAE interaction <10, from which the top sequences ranked by ascending pAE interaction were selected for yeast display. For campaigns that initially failed to yield binders, we additionally applied fold-conditioning to previously validated scaffolds to improve structural stability. In the BindCraft workflow^7^, we hallucinated binder backbones (typically 50–65 residues) directly from the same antigen structures without pre-specifying hotspots, applied the default multimer filters, and selected the top ~100–125 filter-passing designs ranked by ipTM after deduplicating by backbone for experimental testing.

### Lead optimization with CARPNN

To optimize our lead binders while preserving most of their functional properties as CARs, we reasoned that diversifying a limited set of residues while maintaining high confidence in silico metrics might help us explore the functional landscape more efficiently. To perform sequence variation on the parental structure, we divided a binder’s residues into interface or non-interface residues on the basis of whether any atoms of the residue are within 4 Å of any atoms in the target structure. Each set of the residues was then mutagenized via SolubleMPNN with a temperature parameter of 0.4, with 2,000 draws each (BCMA) or 5,000 draws each (CD19, CD22). A Levenshtein distance cut-off of 5 (BCMA) or 10 (CD19, CD22) was then applied to ensure the mutated binder sequences are sufficiently different in sequence composition from the parental binder. After the sequences were selected, these sequences were then folded against the target antigen using a BindCraft-based Boltz filtering pipeline. In particular, we folded the candidate binder sequences against the target antigen sequences using Boltz1 with 5 diffusion samples and 10 recycles. Key metrics such as pLDDT, pAE, ipTM and other biophysical metrics were then calculated and averaged across the 5 models. Filter terms used in BindCraft were then converted to their closest equivalent terms reported by Boltz1. For example, the cut-off used for ‘Average_pLDDT’ in BindCraft was used to define the cut-off for ‘Average_complex_plddt’ in Boltz1. To ensure the backbone structures are controlled for between binders, a filter was applied to remove binders whose alpha carbon RMSD is more than 1Å away from the parental backbone.

After filtering for high-quality in silico binders, we diversified on 4 binder attributes to maximize observing attributes that correlate with CAR activity. For interface mutagenized binders, we performed *k*-means clustering on the binders by the number of interface hydrogen bonds formed, as well as dSASA against the target antigen. Twelve cluster representatives were then selected for experimental testing. For non-interface mutagenized binders, we employed the same strategy and clustered them by the total number of hydrophobic residues and the total charge of the binder at pH 7.4, and similarly selected the cluster representatives for experimental testing.

### Yeast surface display

Yeast display constructs were generated using the pETcon(−) vector (Addgene, 41522), which contains a GAL1 promoter for inducible minibinder expression and TRP1 as the auxotrophic selection marker. The pETcon(−) backbone was linearized using the restriction enzymes NdeI (NEB, R0111S) and BamHI (NEB, R0136S). Each design campaign was synthesized as an oligonucleotide pool (oPool, Integrated DNA Technologies) containing 5′ and 3′ homology arms to the pETcon(−) backbone, which had been linearized with NdeI and BamHI.

For each oPool campaign, 100 ng of DNA was PCR amplified and purified using the QIAquick PCR Cleanup kit (Qiagen, 28104). The amplified oPool and the linearized backbone were combined and concentrated using the DNA Pellet Paint (Millipore, 70748–3). *S. cerevisiae* strain EBY100 was transformed with 4,000 ng of PCR-amplified insert containing pETcon(−) backbone homologous arms and 1,000 ng of linearized vector using the Frozen-EZ Yeast Transformation II kit (Zymo Research, T2001). Successfully transformed clones were selected using SDCAA growth medium for 48 h at 30 °C.

For minibinder library display, yeast cells were transferred into SGCAA medium at a density of 0.3–0.5 × 10^7^ cells per ml and incubated for 16 h at 20 °C with shaking at 200 r.p.m. Cells were collected by centrifugation at 4,000 × *g* for 1 min and washed once with PBS-B buffer (PBS supplemented with 0.1% (w/v) BSA). For binding assays, induced yeast cells were incubated with biotinylated antigen for 1 h at room temperature, washed twice with PBSF, and stained with a cocktail of APC–streptavidin and anti-Myc–FITC antibodies.

Yeast libraries displaying minibinders were induced for 16 h at 20 °C in SGCAA medium with shaking at 200 r.p.m. Following induction, cells were washed twice with PBS-B and incubated with Biotin-Binder Dynabeads (Thermo Fisher, 11047) without antigen to deplete clones exhibiting non-specific bead binding. The supernatant containing unbound yeast was collected and incubated with Dynabeads pre-conjugated to biotinylated target antigen for positive selection. After incubation, bead–yeast complexes were washed five times with PBS-B to remove unbound cells and resuspended in 50 ml SDCAA medium for outgrowth and enrichment of antigen-binding clones.

### Recombinant binder expression and purification

Minibinder sequences were cloned into a modified pBAD bacterial expression vector using Gibson assembly, incorporating a C-terminal 6× His tag. Each construct was transformed into chemically competent *E. coli* BL21 (DE3) cells (Invitrogen, C600003) and plated on LB-agar containing 100 μg ml^−1^ carbenicillin. Single colonies were inoculated into 50 ml of Terrific Broth (TB) and cultured overnight at 37 °C. The following day, 2 ml of the starter culture was transferred into 1 l TB and grown at 37 °C until reaching an optical density at 600 nm (OD_600_) of 0.6–0.8. Arabinose (20%; Fisher Scientific, AAA1192130) was then added to a final concentration of 0.2% (w/v) to induce minibinder expression, and cultures were incubated overnight at 20°C with shaking. The following day, cells were collected by centrifugation at 3,220 × *g* for 10 min, resuspended in lysis buffer supplemented with protease inhibitor tablets (Pierce, A32963) (50 mM Tris-HCl (pH 8.0), 300 mM NaCl, 15 mM imidazole, 0.25% NP-40, 0.25% Triton and 10% glycerol) and sonicated for 1 min at 1 sonication per 3 s (3× with a 3 min break in between sonications). Whole-cell lysates were clarified by centrifugation at 28,880 × *g* for 1 h at 4 °C. Minibinder proteins were purified from the supernatant using His60 Ni Superflow Resin (Thermo Fisher, A50589) and washed with buffer (50 mM Tris-HCl, 300 mM NaCl, 40 mM imidazole, 10% glycerol), followed by elution with buffer (50 mM Tris-HCl, 300 mM NaCl, 250 mM imidazole, 10% glycerol) using a gravity-flow column. Eluted proteins were purified by size-exclusion chromatography on a Superdex 75 Increase 10/300 GL column (Cytiva, 29148721) in PBS (pH 8.0).

### Biolayer interferometry

Binding kinetics were measured using the Gator Pro Biolayer Interferometry (BLI) system (Gator Bio). Streptavidin biosensors (GatorBio, 160002) were equilibrated in PBS-B (PBS supplemented with 0.2% BSA) and loaded with biotinylated target antigen (20 nM) during the loading phase. Following a brief baseline step, biosensors were transferred into wells containing minibinder proteins at increasing concentrations to monitor association (400 s), then into buffer-only wells to measure dissociation (600 s). Sensorgrams were reference subtracted and fitted using the Gator Pro analysis software to derive kinetic parameters (*k*_on_, *k*_off_) and equilibrium dissociation constant (*K*_d_).

### CAR Jurkat cell generation

The CAR lentiviral backbone (PWL003) was constructed with a CD8a signal peptide and hinge region, followed by a de novo designed minibinder sequence flanked at the N terminus by a Myc-epitope tag for detection driven by an EF1α promoter within a second-generation lentiviral cassette. The intracellular domains consisted of 4–1BB and CD3ζ to provide co-stimulatory and activation signalling, respectively.

For engineering Jurkats, lentiviral particles were generated following the previously described HEK293T transfection protocol. For transduction, 5 × 10^5^ Jurkat cells were seeded in 2 ml RPMI medium per well of a 6-well plate, and three viral titres (100–800 μl of freshly collected lentiviral supernatant) were applied. Polybrene (4 μg ml^−1^) was added, followed by spin inoculation at 800 × *g* for 30 min. Transduction efficiency was assessed at 60 h post infection by flow cytometry as the percentage of GFP^+^ cells. CAR Jurkat populations exhibiting 25–55% GFP positivity were selected for subsequent experiments.

### CAR Jurkat co-culture CD69 activation assays

CAR Jurkat (CAR J) cells were selected on the basis of GFP positivity, counted and centrifuged at 300 × *g* for 5 min. Cells were resuspended in RPMI medium at 5 × 10^5^ live GFP^+^ cells per ml. Since GFP positivity varied across transductions, the total number of cells used for resuspension was adjusted accordingly to achieve this target concentration of GFP^+^ cells. Target cell lines, either expressing or lacking the cognate surface antigen, were also counted and adjusted to 5 × 10^5^ cells per ml in RPMI medium. All cell lines in this work were purchased from ATCC in the past 2 years. Cell line identities were confirmed via STR profiling via ATCC. Flow cytometry validation of CD19, BCMA and CD22 was performed and corroborated expected profiles based on RNA-seq profiles.

Co-cultures were established in 96-well u-bottom plates by seeding 5 × 10^4^ GFP^+^ CAR J cells with 5 × 10^4^ target cells per well. For co-cultures with variable effector-to-target (E:T) ratio, 2.5 × 10^4^ GFP^+^ CAR J cells were seeded in each well, and the number of target cells was adjusted accordingly. Control wells containing CAR J cells alone or target cells alone were included. Co-cultures were incubated for 16 hours at 37 °C in the incubator.

After the incubation, the co-culture plate was centrifuged at 300 × *g* for 5 min. The supernatant was discarded, and the cells were washed with 200 μl of PBS containing 0.5% BSA. The cells in each well were then stained with 100 μl of the antibody cocktail (diluted in PBS containing 0.5% BSA) consisting of 0.1 μl LIVE/DEAD Fixable Near IR (780) stain (Invitrogen, L34992), 0.5 μl anti-CD3 APC conjugated antibody (Biolegend, 300312), 0.5 μl anti-CD69 PE-Cy7 conjugated antibody (Biolegend, 310912) and 0.5 μl anti-target antigen fluorophore conjugated antibody. After 30 min of incubation at 4 °C, the cells were washed once and resuspended in 150 μl PBS with 0.5% BSA. The 96-well plate containing the cell samples was then processed using an Attune CytPix Flow Cytometer and CytKick Autosampler (Invitrogen, A42901).

### Recombinant antigen binding assay for CAR Jurkats

CAR Jurkat cells (25,000 per well) were seeded in 96-well plates in 100 μl RPMI-1640 medium supplemented with 10% fetal bovine serum and incubated overnight (16 h) at 37 °C. The following day, cells were washed once with 1× PBS-B (0.25% BSA) and incubated with varying concentrations of biotinylated recombinant antigen BCMA (Sino Biological, 10620-H40H-B), CD22 (Sino Biological, 11958-H49H-B) or CD19 (Sino Biological, 11880-H49H-B) for 1 h at room temperature. After incubation, cells were washed twice with 1× PBS-B and stained with anti-streptavidin–APC conjugated antibodies (BioLegend, 405207) for 30 min at 4 °C. Cells were then washed twice and resuspended in 150 μl PBS-B for flow cytometry analysis.

### Primary CAR T assays

T cells were isolated from Stem Cell Leukopacks (Stem Cell, 200–0092) using EasySep Human T Cell Isolation kit (Stem Cell, 17951) for three different human donors. Upon thawing, cells (day 0) were resuspended in Immunocult (Stem Cell, 10981) supplemented with 0.1 μg ml^−1^ IL-2 (Stem Cell, 78036) and activated with ImmunoCult Human CD3/CD28 T Cell Activator (Stem Cell, 10971). Cells were maintained at a concentration of 0.25 × 10^6^ ml^−1^. On day 2, concentrated lentivirus was added to cells, and cells were spin infected at 800 *g* for 45 min. On day 5, DAPI-stained T cells were sorted for GFP^+^ cells using the SH800S Cell Sorter.

### Cytokine measurements via ELISA

On day 11, cells were collected to set up co-cultures; for flow: 50,000 each of target and effector cells were resuspended in 200 μl per well of a 96-well plate, incubated for 16 h; for IncuCyte: 10,000 each of target and effector cells were resuspended in 200 μl per well of a 96-well plate. Cells in the flow co-culture plate were spun down, and supernatant was collected and frozen at −80 °C in 10 μl aliquots for ELISA assays, and cells were processed for CD69 activation analysis via flow cytometry.

For ELISA, the supernatant was prepared according to manufacturer instructions. Kits and input of supernatant used were IFNɣ (Biolegend, 430101), IL-2 (Biolegend, 431801), Granzyme B (Invitrogen, BMS2027–2) and TNF (Biolegend, 430201). Cytokine readouts were quantified using a Varioskan LUX Multimode Microplate Reader (VLBLATD2, Thermo Scientific) at an absorbance of 450 nm.

### CAR T mediated cytotoxicity assays

For live-cell cytotoxicity assays, cells were co-seeded in 200 μl of medium per well in an IncuCyte ImageLock 96-well microplate (Sartorius, BA-04856). Target cells were stably labelled with mCherry, and CAR T cells were labelled with GFP. Live-cell imaging was performed using the IncuCyte system, acquiring 4 fields per well every 4 h for 72 h. Cell viability was quantified as the total red object integrated intensity (RCU × μm^2^ per image) normalized to the initial time point (0 h).

### Repetitive stimulation, absolute proliferation and functional killing assays

To assess CAR T durability under chronic antigen exposure, 1 × 10^5^ GFP^+^ CAR T cells were co-cultured with 8 × 10^5^ mCherry^+^ RPMI-8226 target cells (1:8 E:T ratio) in 2 ml of Immunocult supplemented with 1% penicillin–streptomycin in a 12-well plate as the initial stimulation. Every 3–4 days, cells were collected and counted by flow cytometry on the Attune CytPix Flow Cytometer using CountBright Plus Absolute Counting Beads (Thermo Fisher, C36995) to determine absolute counts of GFP^+^(CAR T) events and quantify CAR T expansion, and stained for the exhaustion markers LAG-3-BV421 (Clone T47–530, BD-Biosciences) and PD-1-APC (Clone J105, eBioscience). Following each count, 1 × 10^5^ CAR T cells were restimulated with 8 × 10^5^ fresh RPMI-8226 target cells to maintain a 1:8 E:T ratio. Stimulations were performed for a total of 4 rounds.

Following the fourth and final stimulation, the functional capacity of repeatedly stimulated CAR T cells was assessed by co-culturing 1 × 10^4^ CAR T cells with 1 × 10^4^ RPMI-8226 target cells (1:1 E:T ratio). Target cell killing was quantified by the loss of mCherry^+^ events over 5 days using the IncuCyte system. Each condition was performed in 3 biological replicates.

### In vivo xenograft efficacy study

Animal work in this paper was reviewed by the Institutional Animal Care and Use Committee of Memorial Sloan Kettering Cancer Center and was approved (Protocol #25–10-007; ‘Tumour control via novel biologics and cell therapies’). Female NOD.Cg-*Prkdc*^*scid*^ *Il2rg*^*tm1WjI*^/SzJ (NSG) mice (6–8-week-old) were purchased from the Jackson Laboratory for all experiments. After a week of acclimation, mice were injected subcutaneously in the right flank with 3 × 10^6^ RPMI-8226 cells stably expressing firefly luciferase, generated by lentiviral transduction with Lenti-luciferase-P2A-Neo (Addgene, 105621) and selection with G418. Once tumours were palpable (~1.5 weeks), 3 × 10^6^ CAR Ts expressing different binders were injected intravenously (i.v.) through the tail vein of each mouse. Treatment arms included vehicle (PBS), untransduced T cells (UTD), C11D5.3 and B5.I0. Tumour bioluminescence of each mouse was imaged weekly via intraperitoneal (i.p.) injection of IVISbrite D-luciferin potassium salt bioluminescent substrate (122799 A) at 3 mg per mouse. At the end of the study, mice were euthanized and blood analysis was conducted to assess CAR T persistence (GFP^+^ detection) via flow cytometry.

### Low effector:target in vivo xenograft efficacy study

To evaluate CAR T efficacy under more limiting effector conditions, the xenograft study was performed following the in vivo xenograft efficacy method described above with the following modifications. Female NSG mice (6–8-week-old) were injected subcutaneously (s.c.) with 3 × 10^6^ of previously described RPMI-8226 luciferase-tagged cells. Once tumours were palpable (~1.5 weeks), 3 × 10^5^ CAR Ts expressing different binders were injected i.v. through the tail vein of each mouse. Treatment arms included vehicle (PBS), untransduced T cells (UTD), C11D5.3, VHH1-VHH2 (binder used in CARVYKTI) and B5.I0. Weekly tumour bioluminescence imaging was conducted to assess tumour growth. For the VHH1-VHH2 group, only 4 mice were used due to low recovery of the transduced CAR T cells for this construct. All other groups had 5 mice.

### Retrospective analyses of CAR campaigns

To infer the on-cell binding affinities of various binders towards their respective target antigen, we performed nonlinear least square (NLS) curve fit of the staining intensities at 0.1 nM, 1 nM, 10 nM and 100 nM concentrations of the target antigen. The estimated affinities were then rescaled using the ratio between the estimated affinity of the positive control and the reported affinity of the positive controls reported in the literature. For curves that had poor fit (*R*^2^ < 0.95), a single point estimate based on the staining intensity at the concentration with the highest dynamic range (BCMA 1 nm, CD19 C1 100 nM, CD19 C2 1 nM, CD22 0.1 nM) was applied instead and rescaled.

To identify the binder design success rate by yeast binding in an unbiased manner, we defined a binder to be YSD enriched at a specific concentration (*X*) if its counts per million (CPM) from deep sequencing exceeded at least 1,000 at the *X* nM sorted pool and it is at least a 2-fold enrichment compared to its CPM in the pre-sorted parental pool.

To calculate the predicted class-I epitope burden, we computed the predicted affinity between all possible 9-mers of the de novo proteins against all supported alleles by NetMHC^48^. The NetMHC class-I burden was then computed as the mean fraction of 9-mer peptides predicted to be strong binders for MHC class-I alleles across all alleles supported by NetMHC4.2.

### Statistical analyses and plotting

All data figures were created using R (v.4.4.1) and ggplot2 (v.3.5.1).

## Extended Data

**Extended Data Fig. 1 |:**
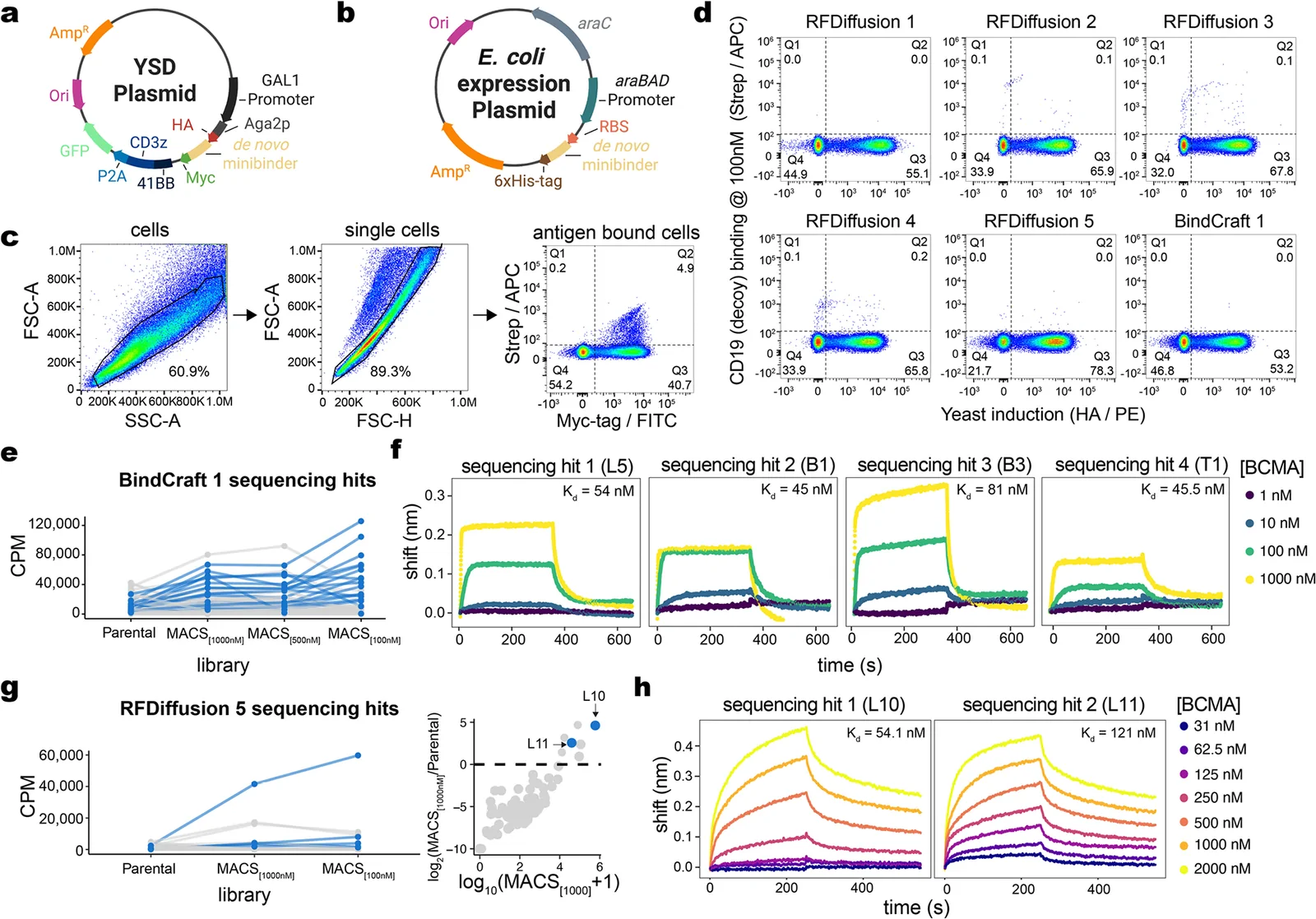
Characterization of lead de novo designed BCMA binders. **a**, Yeast surface display plasmid encoding a de novo minibinder flanked by an N-terminal HA^−^tag and a C-terminal Myc-tag under a GAL1 promoter. **b**, *E.coli* protein expression plasmid encoding de novo minibinder flanked by a 6xHis-tag for purification under a araBad promoter. **c**, Representative gating strategy used during yeast display campaigns to identify binder hits. **d**, Decoy binding of designed binder campaigns against CD19 antigen (100 nM), demonstrating variable levels of off-target reactivity. **e**, Enrichment of individual binders from BindCraft1 (BC1) across successive rounds of magnetic cell sorting (MACS) at decreasing concentrations, demonstrating progressive selection of high-affinity binders. **f**, BLI curves and estimated Kds for additional sequencing hits from the BC1 campaign. **g**, Enrichment of individual binders (L10 and L11) from the RFdiffusion 5 campaign across increasing rounds of MACS. **h**, Biolayer interferometry (BLI) curves and dissociation constants (Kd) values for L10 and L11 binding to BCMA antigen (100 nM) from the RFdiffusion 5 campaign. All BLI and sequencing libraries are individual replicates.

**Extended Data Fig. 2 |:**
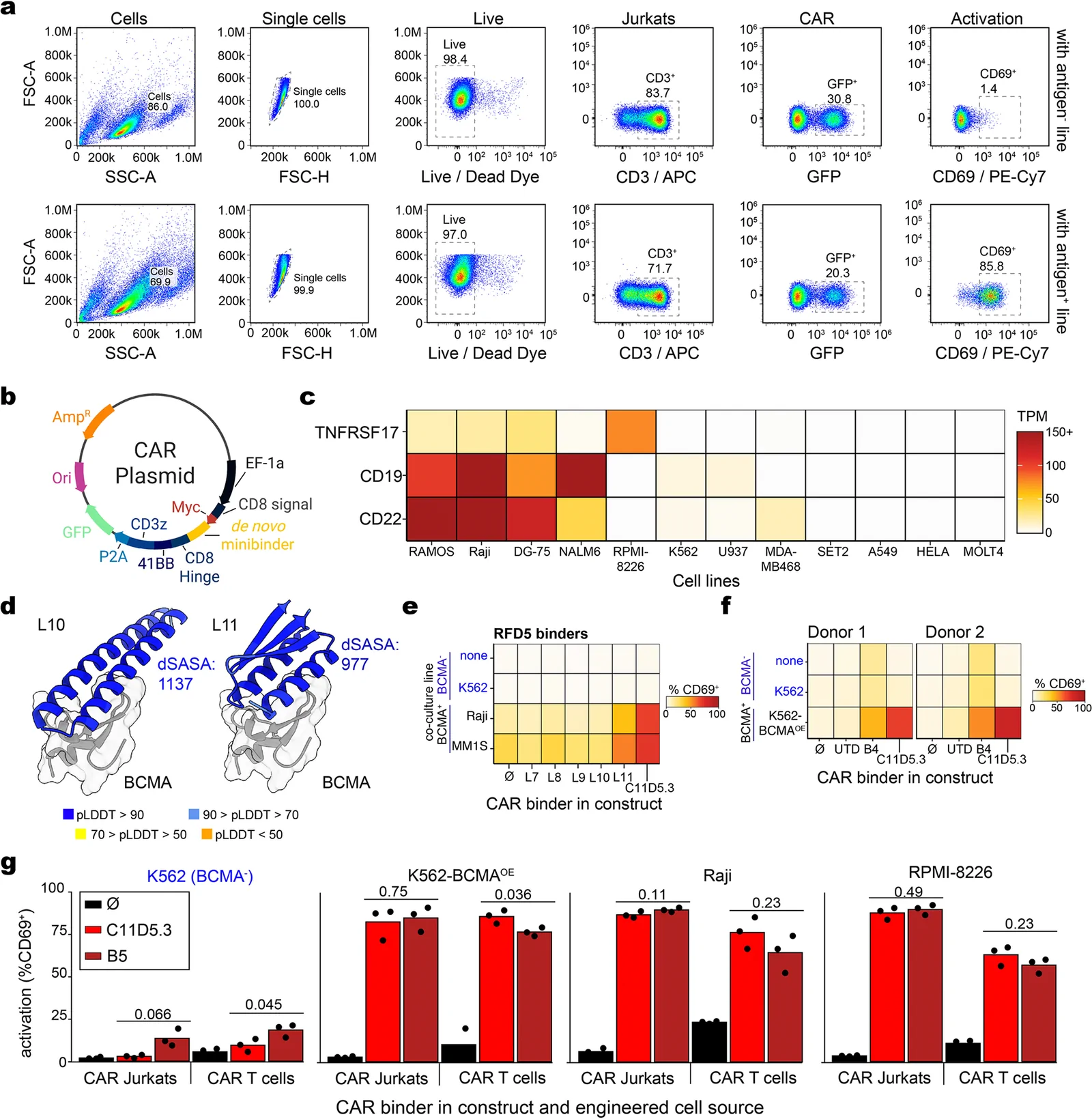
Characterization of lead de novo designed BCMA binders. **a**, Representative gating strategy for CAR Jurkat and CAR T cell co-culture assays used to quantify proportion of cells with CD69 activation. **b**, CAR construct used in CAR Jurkat and CAR T assays containing an EF1α promoter, CD8 signal peptide, de novo minibinder, CD8 hinge, 4–1BB domain, P2A sequence, and GFP reporter. **c**, RNA expression of target genes across cell lines used in this study. TPM: transcript per million**. d**, Predicted structures of two de novo binders designed by RFdiffusion. Binders are colored by per-residue AlphaFold2 predicted Local Distance Difference Test (pLDDT) metrics. **e**, CAR Jurkat co-cultures with RFdiffusion designed L7-L11 BCMA^−^specific de novo minibinders with BCMA^−^ and BCMA^+^ cancer cell lines. **f**, % CD69 activation of CAR Jurkat cells expressing BindCraft1 campaign designed binder (B4) co-cultured with K562 target cells (BCMA^−^ and BCMA^+^). **g**, %CD69 activation was quantified for CARs expressing no binder (Ø, black), C11D5.3 (red), or B5 minibinder (dark red), in either Jurkat cells or primary T-cells in co-cultures with BCMA^−^ and BCMA^+^ cancer cell lines, comparing Jurkat and primary CAR T responses within each binder condition. *P* values calculated using two-sided Student’s *t*-test. All groups show three independent donor replicates.

**Extended Data Fig. 3 |:**
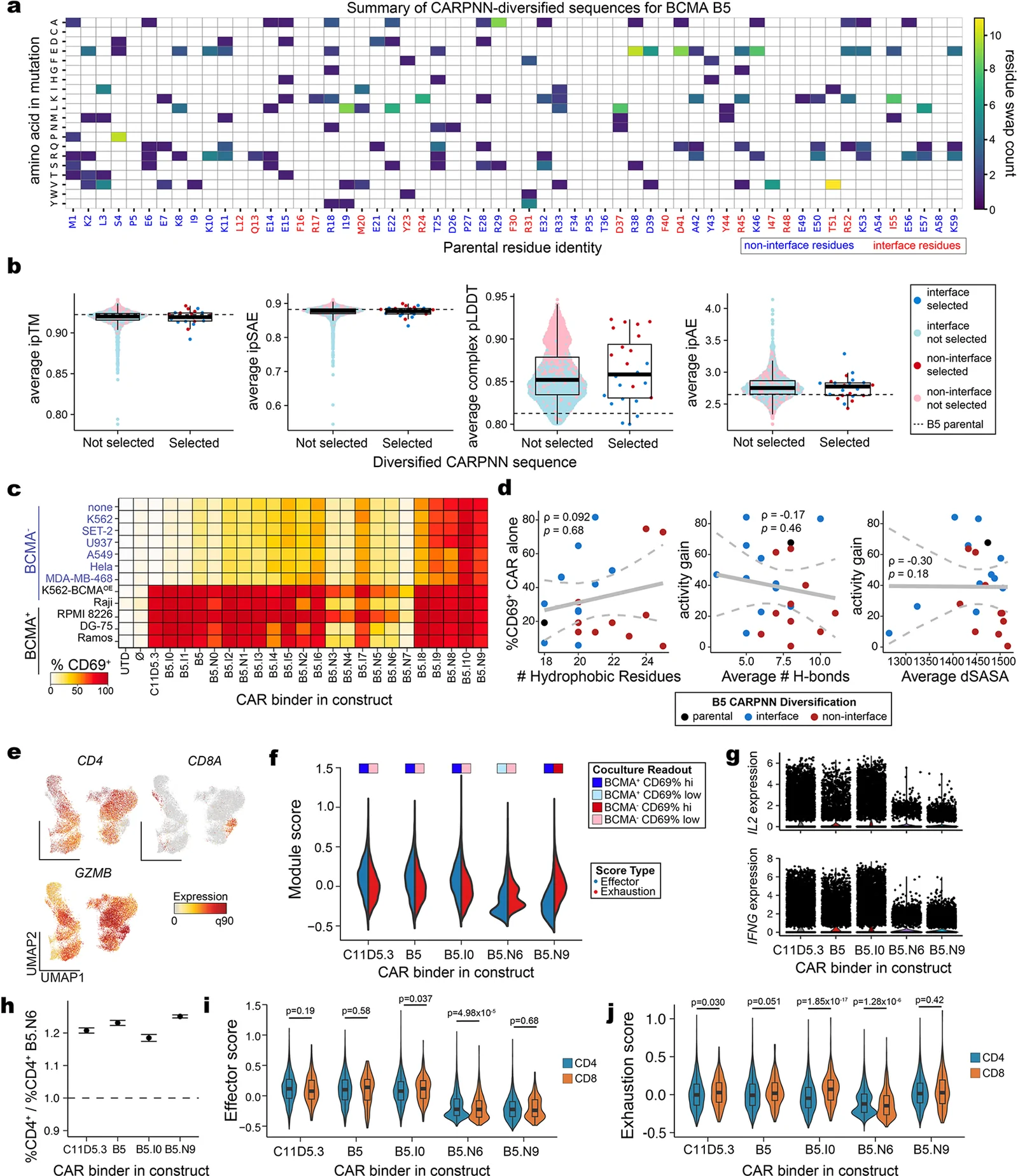
Characterization of the BCMA evolution campaign. **a**, Summary of mutations introduced to each of the diversified BCMA binders relative to the parental B5 binder. **b**, Design metrics of candidate and selected binders from sequence diversification of the BCMA binder (*n* = 2,000). The parental B5 value is noted in the dashed line in each panel. Boxplots: center line, median; box limits, first and third quartiles; whiskers, 1.5× interquartile range. **c**, Summary of diversified sequences as CAR-Jurkats in co-cultures with variable cell lines to assess potential off-target activation and CAR specificity. **d**, Association between attributes diversified during the CARPNN mutagenesis experiment and the CAR attributes they were hypothesized to influence. *P* value and point estimate are reported from a two-sided Spearman correlation test. **e**, Marker gene expression across CAR T cells. **f**, Effector and exhaustion module score across CAR T cells and their relative CD69% in coculture experiment. Module genes were the same as previously used^56^. **g**, Single-cell expression of key effector genes across variable CAR T binders. **h**, Comparison of normalized CD4^+^ cell proportions between CAR T cells against the unreactive binder B5.N6 (*n* = 38,606). **i**, Comparison of effector module score in CD4^+^ CAR T cells. **j**, Comparison of exhaustion module score in CD4^+^ CAR T cells. *P* values are calculated from two-sided Wilcoxon rank sum tests (*n* = 22,184; asymptotic p-value calculation). Boxplots: center line, median; box limits, first and third quartiles; whiskers, 1.5× interquartile range.

**Extended Data Fig. 4 |:**
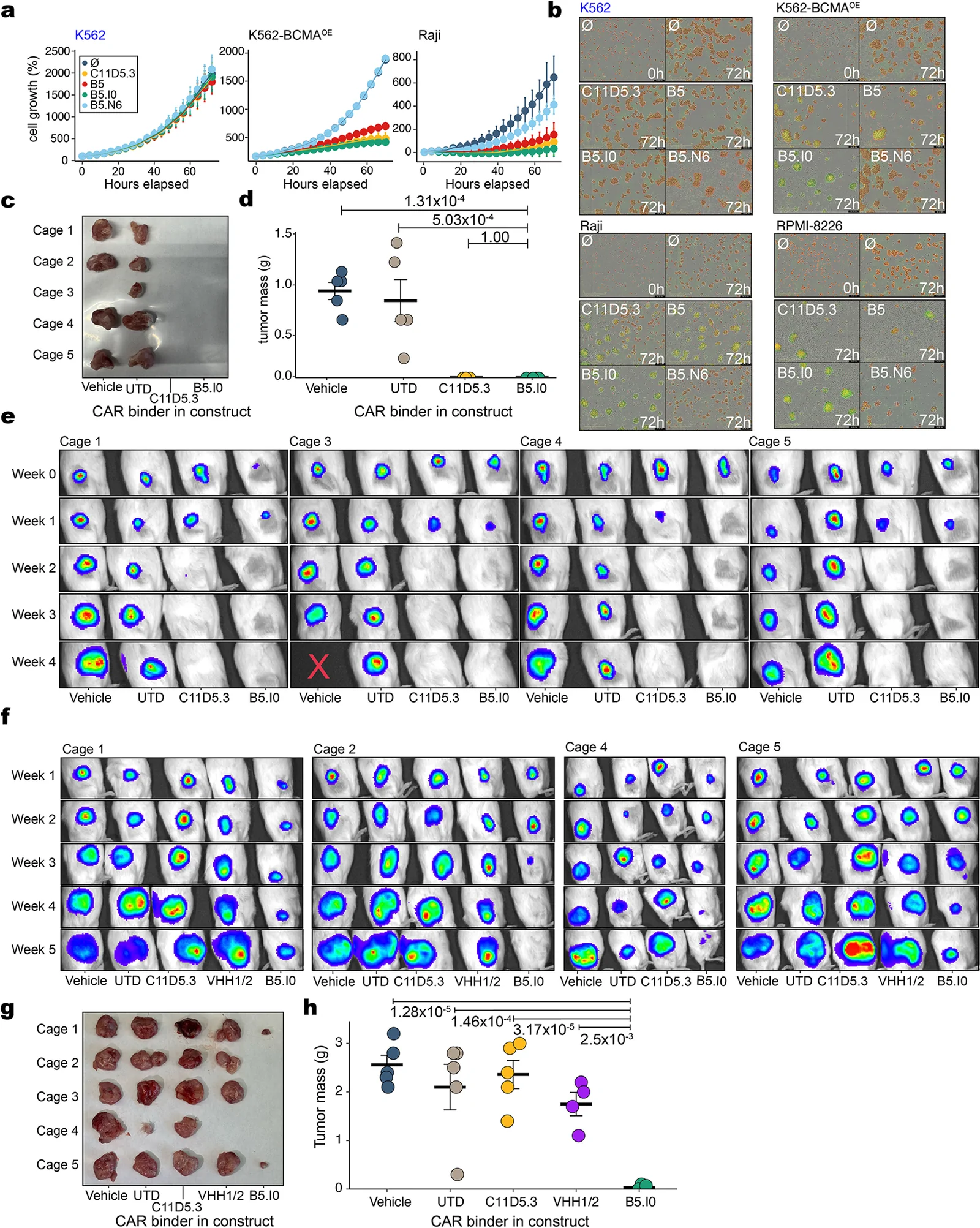
Additional in vivo evaluation of de novo minibinder CAR T antitumor activity. **a**, Killing curves measuring tumor cell growth (% intensity) over 72 h of co-culture with selected CAR constructs against K562 (BCMA^−^), K562-BCMA^OE^(BCMA^+^) and Raji (BCMA^+^) target cells at an E:T ratio of 1:1. Data presented as mean ± SEM. n = 3 independent donors. **b**, Representative IncuCyte images at 0 h and 72 h for each CAR construct co-cultured with the indicated target cell lines. Representative images of three replicates, each with similar results. **c**, Representative images of excised tumors from each treatment arm at endpoint. **d**, Tumor mass (g) at endpoint across four treatment arms. Statistical significance was assessed by one-way ANOVA followed by Dunnett’s multiple comparisons test against B5.I0. **e**, Representative bioluminescence images of mice from cages 1,3, 4, and 5 over four weeks post-treatment. X denotes mouse found dead one day prior to scheduled endpoint imaging (presumed tumor burden). **f**, Representative bioluminescence images from the high tumor burden model (E:T 1:10) across Cages 1, 2, 4, and 5. **g**, Excised tumors at endpoint from **f**. Images are all of tumors, experiment was not **h**, Tumor mass (grams) at endpoint from tumors shown in **f**. Statistical significance was assessed by one-way ANOVA followed by Dunnett’s multiple comparisons test against B5.I0.

**Extended Data Fig. 5 |:**
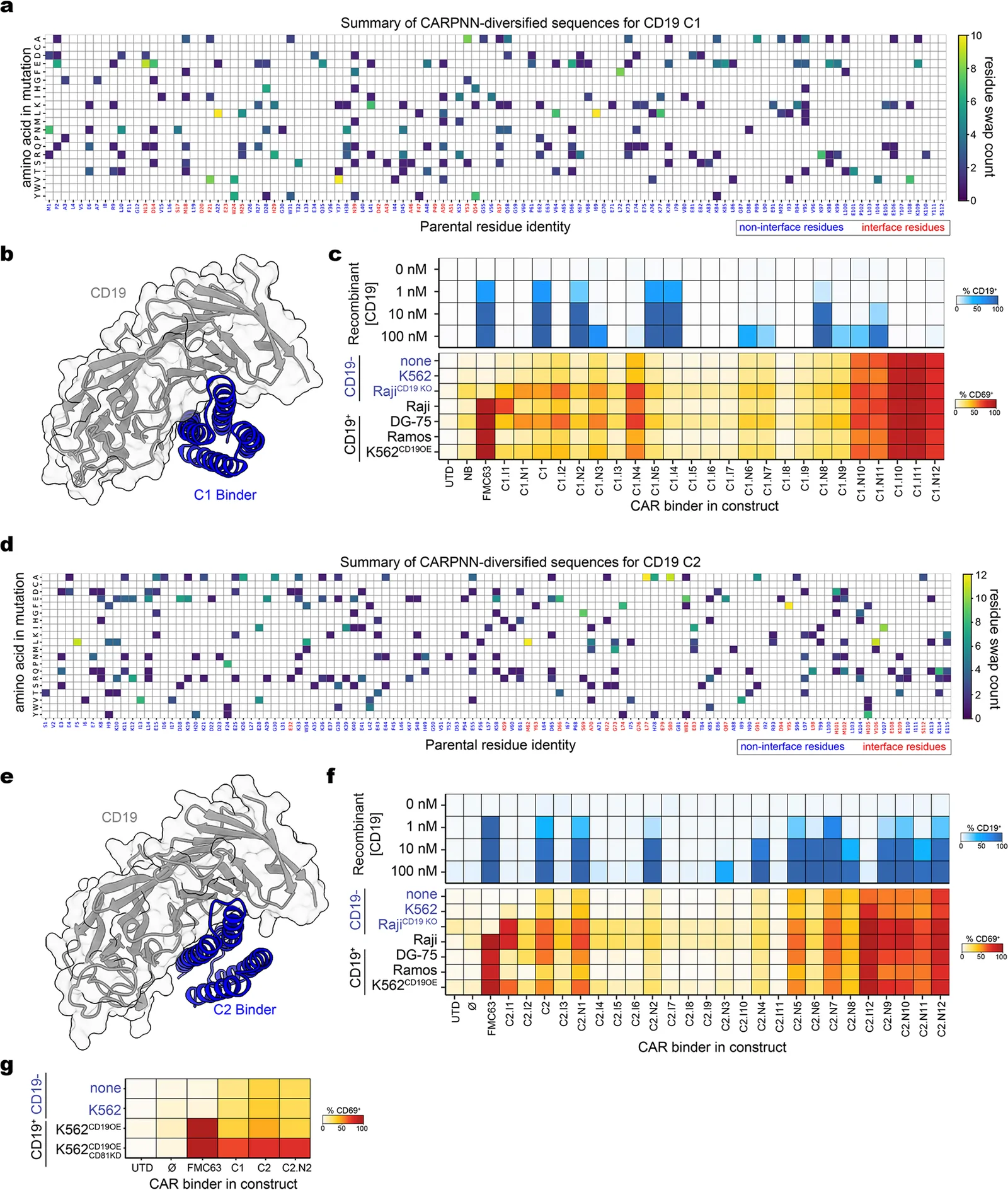
Characterization of the CD19 evolution campaign. **a**, Summary of mutations introduced to each of the CARPNN diversified CD19 C1 binder. Red residue index denotes interface residues while blue index denotes non-interface residues. **b**, Predicted structure of the C1 binder in complex with CD19. **c**, Heatmap of the mutagenized C1 binder variants tested as CARs in co-cultures. Upper panel (blue) displays CAR binding across the indicated antigen concentrations. The lower panel (red) reports %CD69 activation in CD19^−^ and CD19^+^ cell lines. **d**, Summary of mutations introduced to each of the CARPNN diversified CD19 C2 binder. Red residue index denotes interface residues while blue index denotes non-interface residues. **e**, Predicted structure of the C2 binder in complex with CD19 **f**, Same heatmap layout as in **c** but shown here for the mutagenized C2 binder variants tested as CARs. **g**, Heatmap of CD69 activity for C1, C2, C2.N2 as CAR constructs co-cultured with K562 (CD19^−^), K562-CD19 (CD19^+^), and K562-CD19^OE^-CD81^KD^ (CD19^+^, CD81^−^).

**Extended Data Fig. 6 |:**
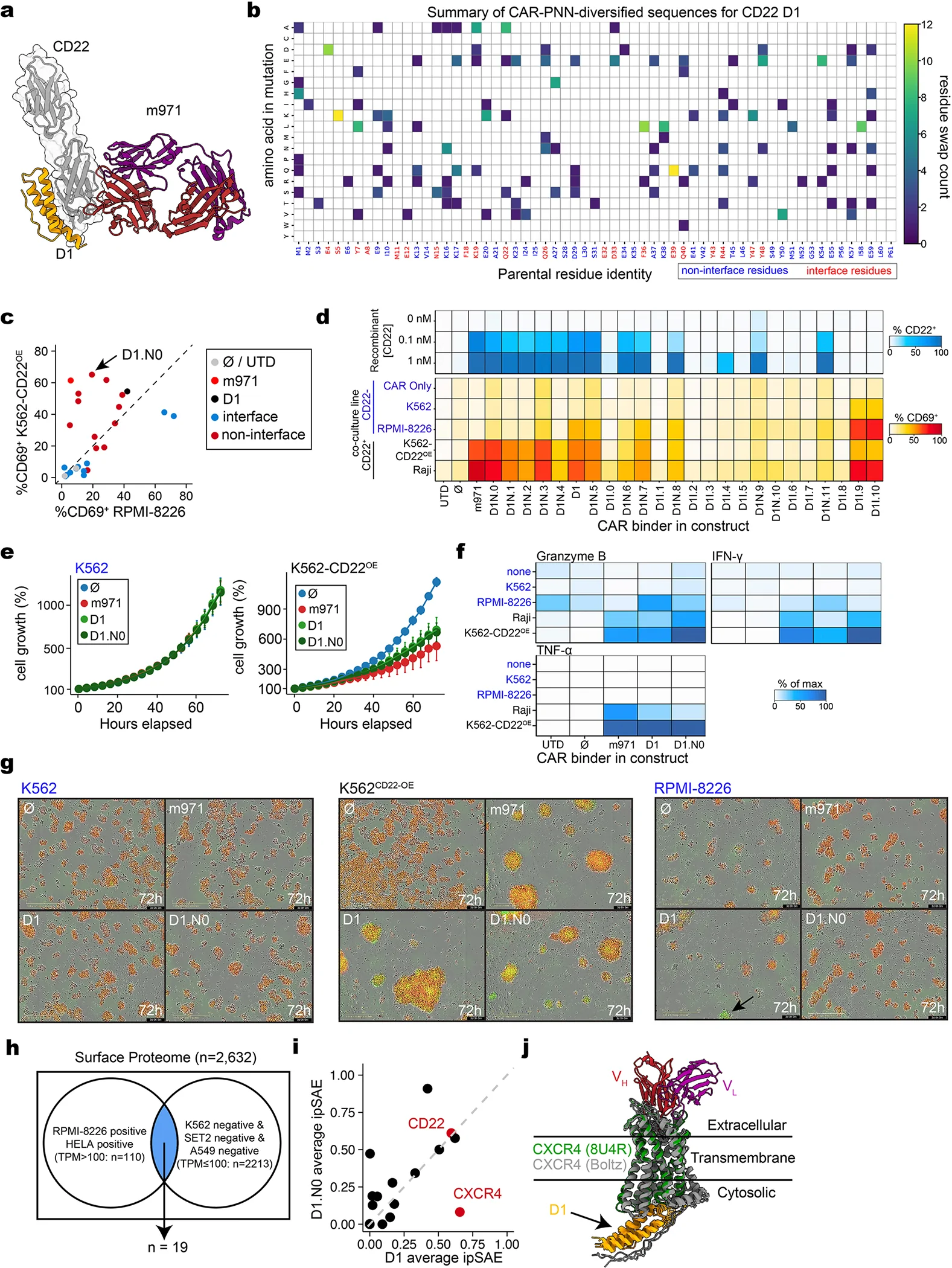
Characterization of the CD22 evolution campaign. **a**, Predicted structure of D1 in complex with CD22 superimposed with m971 in complex with CD22 (PDB: 7O52)**. b**, Summary of mutations introduced to each of the CARPNN diversified CD22 D1 binder. Red residue index denotes interface residues while blue index denotes non-interface residues. **c**, Comparison of CAR activation of the evolved CD22 D1 binders in CD22^−^ RPMI 8226 cell lines and CD22^−^overexpressing K562 cell lines. **d**, Summary of diversified sequences from antigen CAR flow (top) and co-cultures with variable cell lines (bottom). **e**, Representative Incucyte killing assays showing the cytolytic activity of CAR T cells expressing either CD22-specific minibinder- or scFv-based receptors. Time-course plots showing normalized red calibrated unit (%RCU) intensity relative to time 0 h for each construct. Data are presented as mean ± SEM of n = 3 different donors. **f**, Cytokine production from CD22-specific CAR T cells in co-cultures with CD22^+^ and CD22^−^ target cell lines. Heatmap shows mean cytokine levels across triplicates, revealing elevated cytokine release specifically in response to CD22-expressing targets, consistent with antigen-specific activation and killing. **g**, Representative images at 0 h and 72 h for NB and at 72 h for each binder condition to illustrate target-cell killing. Green fluorescence denotes CAR T cells, and red fluorescence denotes the corresponding target cell line. Representative images of three replicates, each with similar results. **h**, Identification of plausible candidates of D1 off-target interaction via subsetting HPA surfaceome and GTEx overlap. **i**, Comparison of average cofolding ipSAE score between parental D1 to all plausible off-target genes and the evolved D1.N0 binder to plausible off-target genes. **j**, Predicted binding site of parental D1 binder towards CXCR4 aligned to a solved structure of CXCR4 (PDB: 8U4R).

**Extended Data Fig. 7 |:**
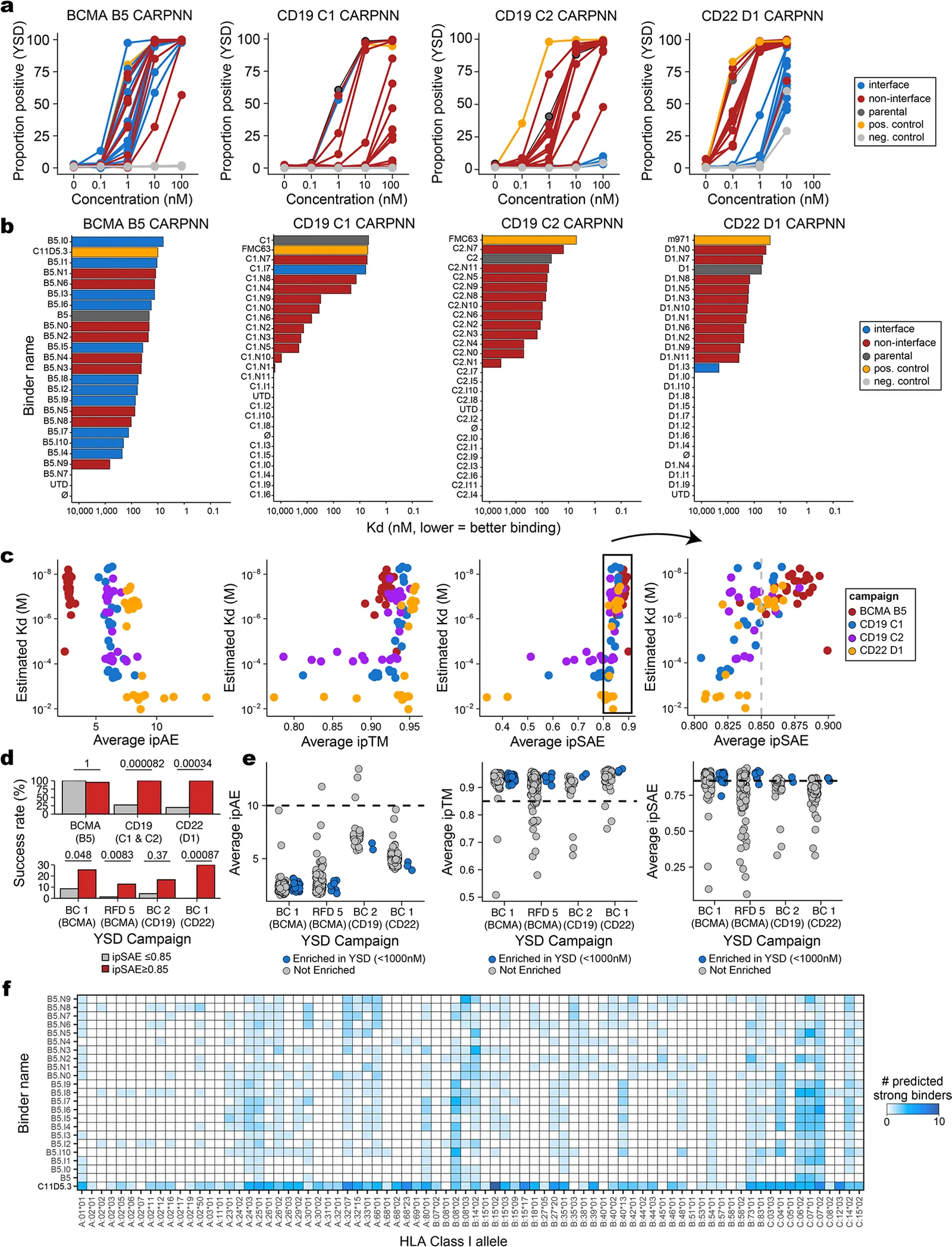
Additional assessment of campaign success rate and binder qualities via in silico metrics. **a**, Staining intensity for CAR Jurkats with antigen at various concentrations. **b**, Binding affinity estimates of all assessed CARPNN diversified binders based on non-linear least squares (NLS) fit of the staining values for good fits (*R*^*2*^ > 0.95) or single point estimates. **c**, Comparison of various protein interaction metrics and their relation to estimated affinity across campaigns. The rightmost panel is a zoomed-in view of the relationship between estimated affinity and average ipSAE. **d**, Comparison of binding success rate of the CARPNN and YSD campaigns using various interaction metrics as cutoffs. CARPNN campaign binding success is defined as having estimated affinity below 1000 nM; YSD campaign binding success is defined as being enriched in the 1000 nM sorted population (CPM > 1000, CPM fold change against parental population > 2). P-value calculated from two-sided Wilcoxon rank-sum test (exact p-value calculation). **e**, Comparison of in silico metrics across yeast surface display (YSD) campaigns where binders were primarily designed using RFdiffusion (RFD) or BindCraft (BC). A sequence is considered YSD enriched at 1000 nM if it has at least 1000 CPM (Counts per Million) at 1000 nM and the CPM is at least two-fold higher than its CPM in the unsorted parental pool. **f**, Comparison of the number of strong HLA-Class I binding peptides of all evolved BCMA binders compared to C11D5.3 (scFv in Abecma) across diverse HLA types.

## Supplementary Material

Supplementary Information

Supplementary Table 3

Supplementary Table 1

Supplementary Table 2

**Supplementary information** The online version contains supplementary material available at https://doi.org/10.1038/s41551-026-01790-9.

## Figures and Tables

**Fig. 1 |:**
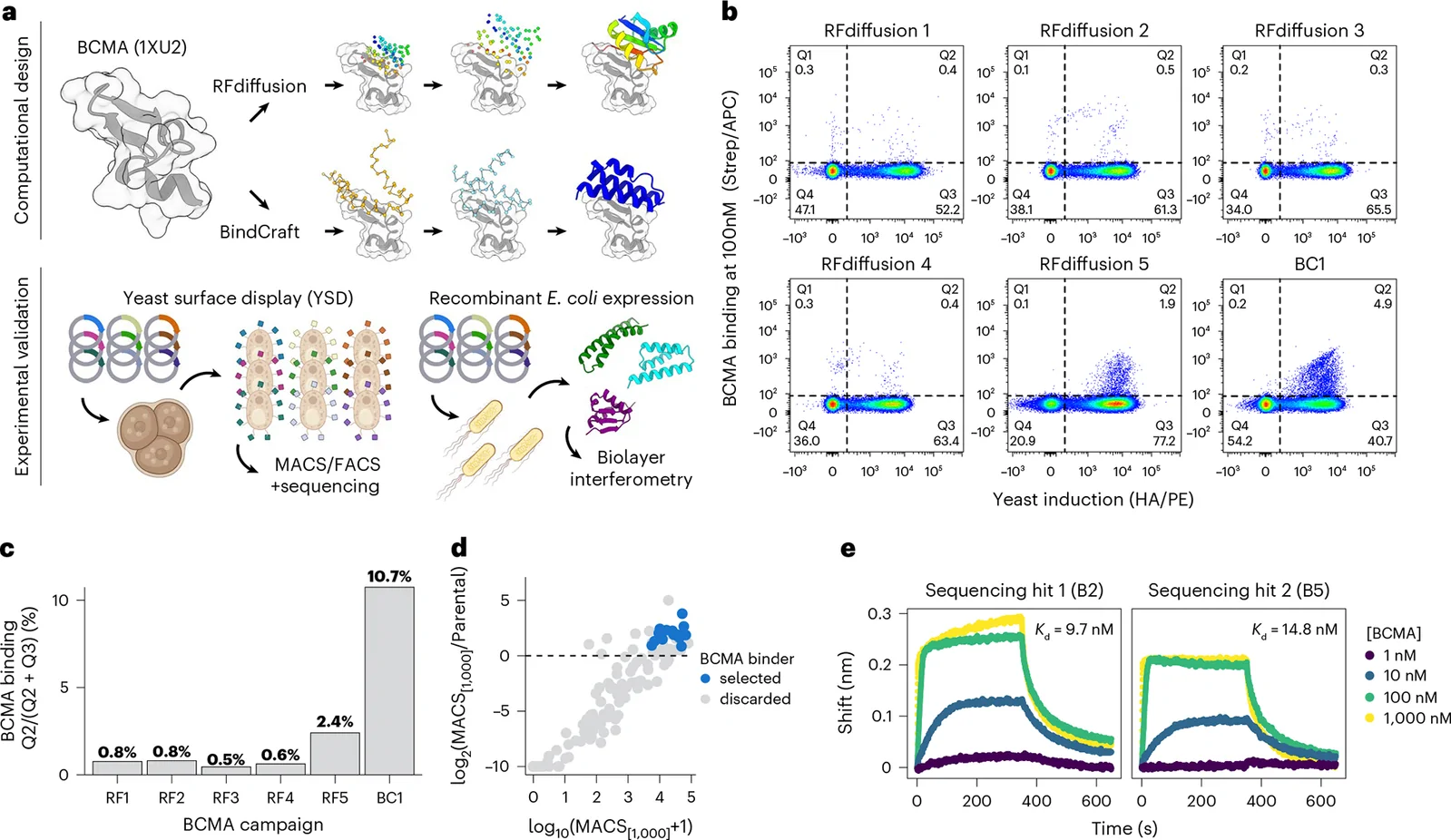
Summary of protein binder development using generative artificial intelligence. **a**, Schematic of computational design (top) and experimental validation (bottom) methods for developing de novo binders targeting BCMA. **b**, Yeast surface display (YSD) results of 6 experimental campaigns of binders for BCMA. **c**, Quantification of YSD enrichment from **b**. **d**, Enrichment of individual binders from the BindCraft1 (BC1) campaign, highlighting enriched binders selected for further validation. **e**, Confirmation of binding for selected binders via biolayer interferometry (BLI). BLI traces are single replicates at 4 shown antigen concentrations.

**Fig. 2 |:**
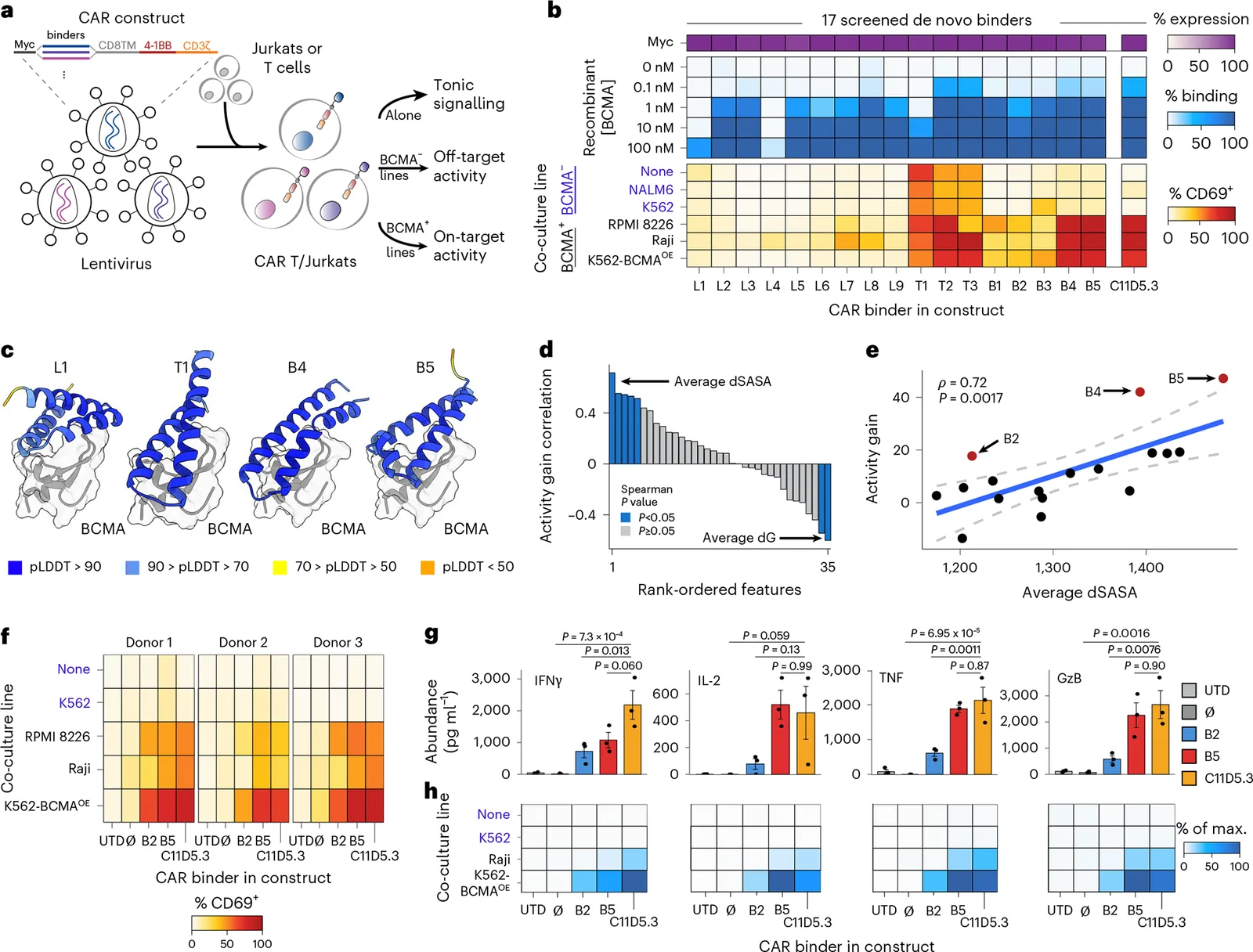
Characterization of de novo designed protein binders as CAR binders. **a**, Schematic of CAR backbone and lentiviral-based systems for assessing CAR activity. **b**, Screen of 17 de novo BCMA binders in heterogeneous conditions compared to the clinical scFv (C11D5.3). Shown is the %CD69^+^ Jurkats among GFP^+^ cells. **c**, Predicted structures of 4 exemplar de novo CAR binders. Binders are coloured by per-residue AlphaFold2 predicted local distance difference test (pLDDT) metrics. **d**, Per-binder metrics associated with variable CAR function, highlighting the top 2 associated with CAR efficacy. Top associations are dSASA and average free energy (dG). **e**, Summary of CAR activation efficacy against per-binder dSASA. *P* value and point estimate are reported from a two-sided Spearman correlation test. **f**, Activation profiles (% CD69^+^) of de novo binders in primary CAR T cells with variable co-culture lines for 3 independent donors. UTD, untransduced; Ø, transduced construct with no binder. **g**, Quantification of cytokine secretion (Granzyme B, IFNγ, IL-2 and TNF) in the donor triplicate for co-culture with RPMI-8226. **h**, Heat map of cytokine secretion from de novo designed binders in primary CAR T cells across multiple target lines. Values represent the mean of *n* = 3 independent donors.

**Fig. 3 |:**
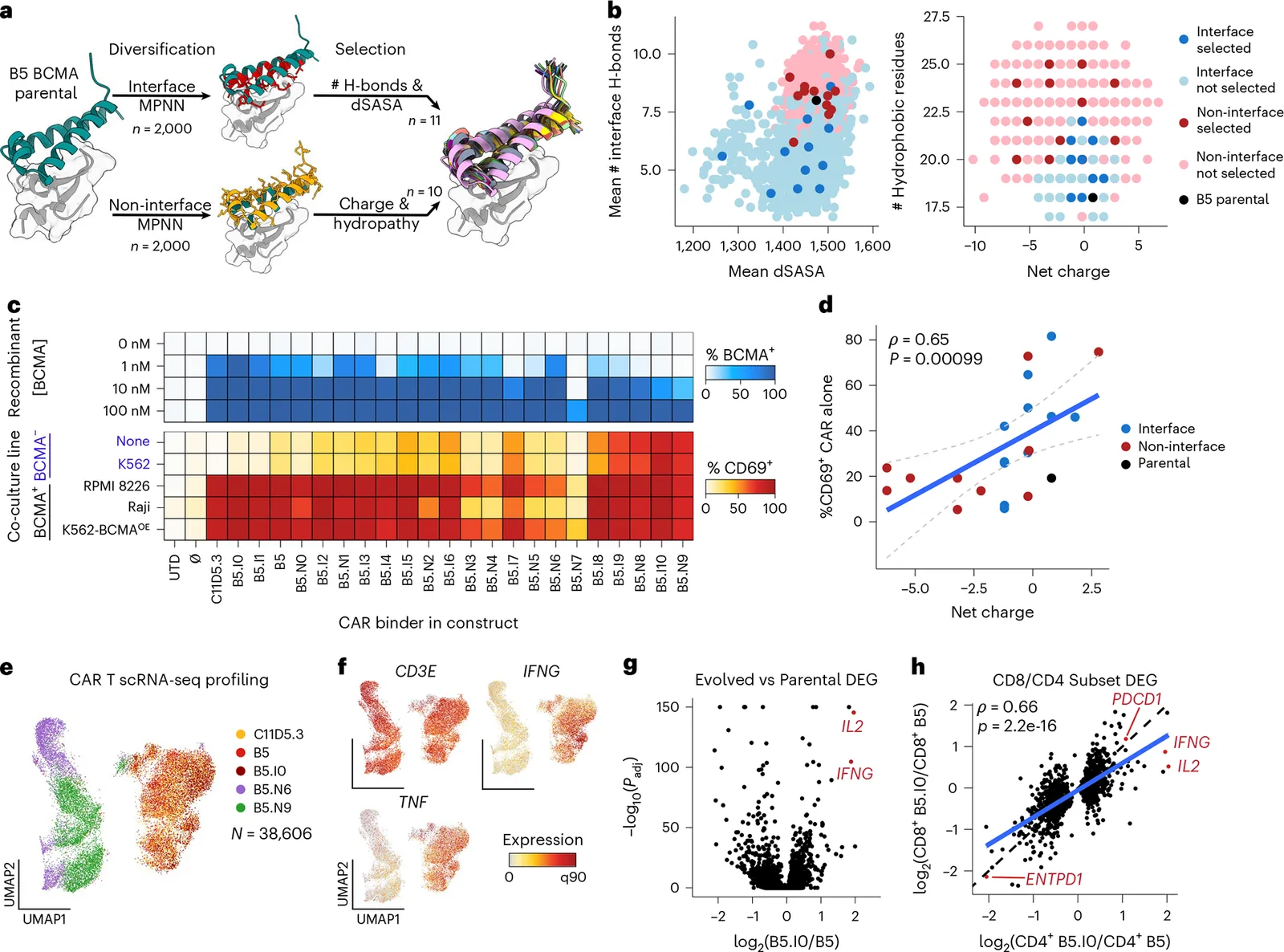
Sequence variation underlies CAR performance. **a**, Schematic of diversifying BCMA binder sequences given a single binder (B5). **b**, Summary of biophysical characteristics of diversified protein sequences, highlighting binders selected for additional CAR testing. **c**, Summary of diversified sequences from antigen CAR flow (top) and co-cultures with variable cell lines (bottom). **d**, Association of binder charge with tonic signalling (data from **c**). *P* value and point estimate are reported from a two-sided Spearman correlation test. **e**, UMAP embedding of 5 CARs profiled via scRNA-seq (*n* = 1 donor). **f**, Marker gene expression across CAR T cells. **g**, Comparison of differentially expressed genes (DEGs) between CAR T expressing evolved (B5.I0) and parental (B5) binder. *P* values were calculated using two-sided Wilcoxon rank-sum tests (exact *P*-value calculation). **h**, Comparison of differentially expressed genes (*P* < 0.05) between evolved (B5.I0) and parental (B5) binder stratified between CD4^+^ and CD8^+^ cells. *P* value and point estimate are reported from a two-sided Spearman correlation test.

**Fig. 4 |:**
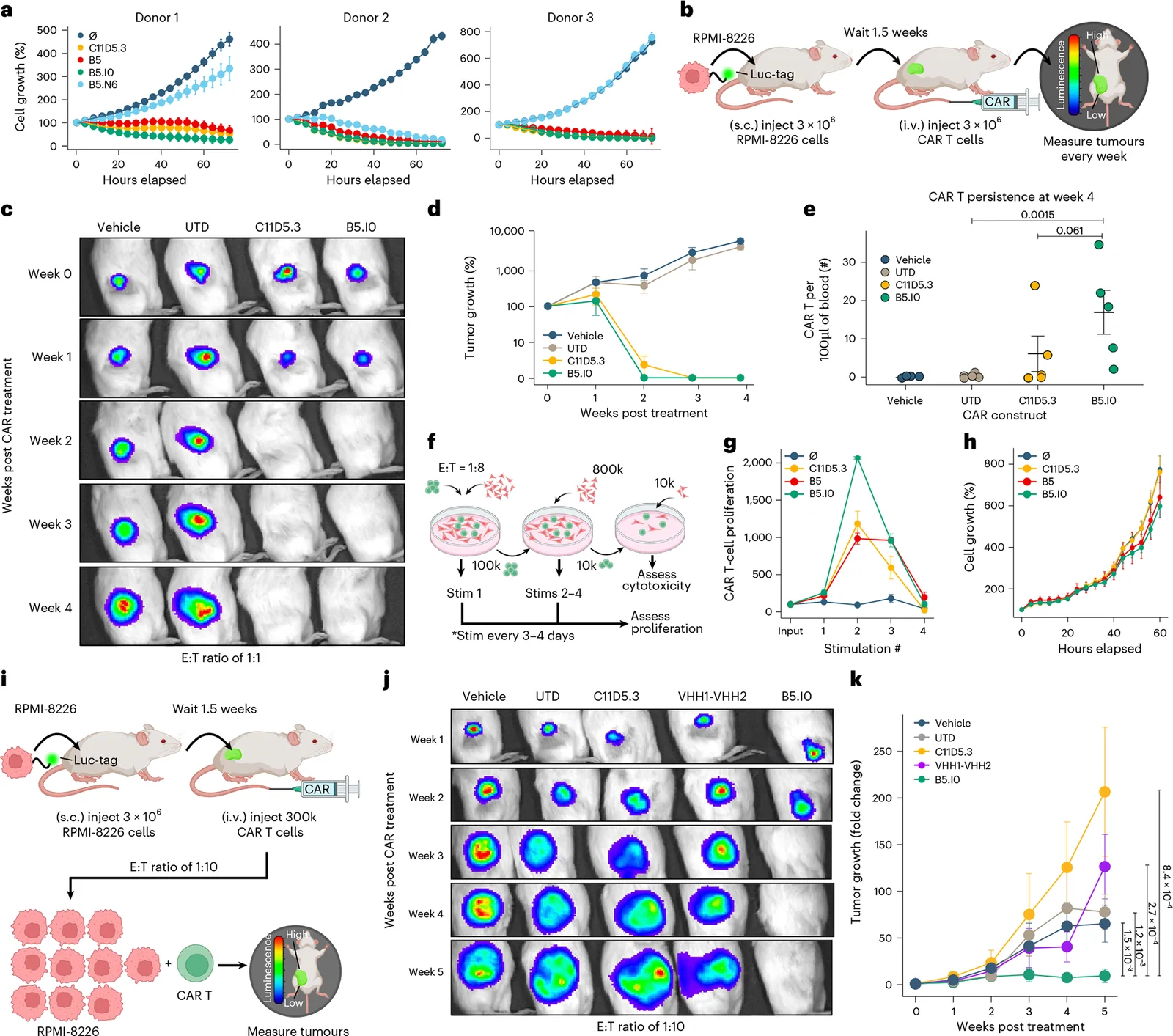
Tumour control of de novo CARs in in vitro and in vivo models. **a**, Killing curves measuring tumour cell growth (% intensity) over 72 h of co-culture with selected CAR constructs against RPMI-8226 target cells at an E:T ratio of 1:1, shown for 3 independent donors. **b**, Schematic of the in vivo xenograft experiment. Luciferase-tagged RPMI-8226 cells (3 × 10^6^) were injected subcutaneously into the right flank of NSG mice, followed by injection of CAR T cells (3 × 10^6^), with weekly tumour burden measured by bioluminescence imaging. Total flux (photons per second) was quantified using Living Image software. The % of tumour growth shows the total flux expressed as a percentage measured for the same animal at week 0. **c**, Representative images of mice from Cage 2. Treatment arms: vehicle, untransduced T-cells (UTD), C11D5.3 or B5.I0. **d**, Quantification of tumour bioluminescence over 4 weeks across 4 treatment arms. Data are presented as mean ± s.e.m. of *n* = 5 mice per group at each timepoint. **e**, Absolute counts of CAR T cells (CD3^+^GFP^+^) in 100 μl of peripheral blood at endpoint. *P* values calculated from a two-sided Wilcoxon rank-sum test (exact *P*-value calculation). **f**, Schematic of the repetitive stimulation assay. CAR T cells were co-cultured with target cells at an E:T ratio of 1:8 for 4 sequential stimulations, after which killing capacity was assessed. **g**, Absolute CAR T-cell counts (GFP^+^) quantified by flow cytometry at each stimulation. Data are mean ± s.e.m., *n* = 3 biological replicates. **h**, Killing capacity after the fourth stimulation, measured as % mCherry^+^ target cell growth over 72 h. **i**, Schematic of a high tumour burden in vivo model at an E:T ratio of 1:10, with tumour burden assessed weekly over 5 weeks. **j**, Representative images from Cage 3. **k**, Quantification of tumour luminescence across 5 treatment arms (vehicle, UTD, C11D5.3, VHH1-VHH2 [CARVYKTI], B5.I0). Data are mean ± s.e.m., *n* = 5 mice per group. Statistical significance at endpoint (week 5) was determined using one-way analysis of variance (ANOVA) followed by Dunnett’s multiple-comparisons test against B5.I0.

**Fig. 5 |:**
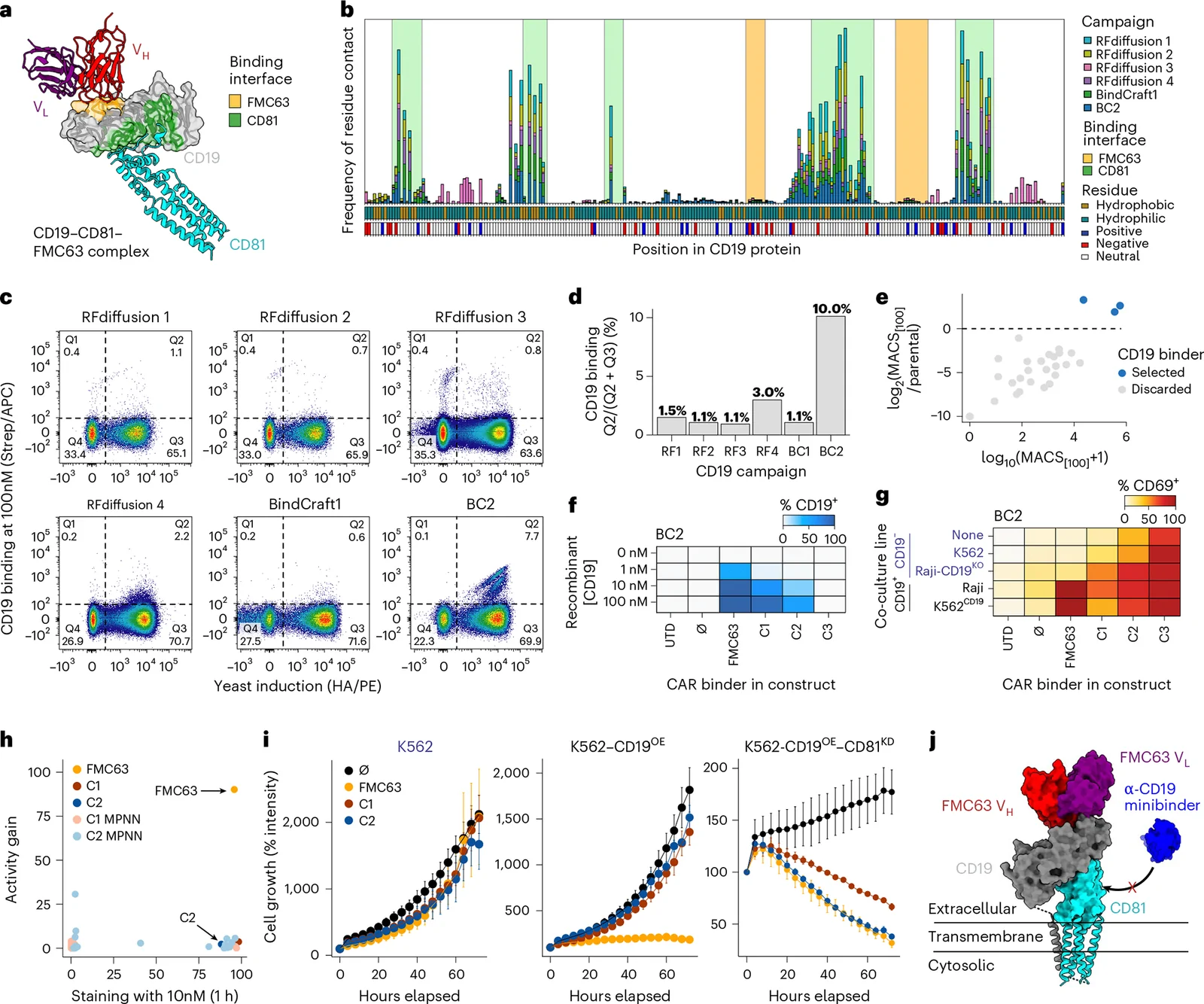
Native binding partners occlude binding epitopes from de novo CD19-directed CARs. **a**, Structural rendering of the FMC63–CD19 complex (PDB 7URV) aligned to the CD19–CD81 complex (PDB 7JIC). The binding surfaces on CD19 for both proteins are highlighted. **b**, Summary of CD19 protein, highlighting residues and binding surfaces. Barplots show the normalized proportion (out of 100 draws) of contacted residues from design campaigns. **c**, Summary of yeast surface display binding of de novo designed proteins against CD19. **d**, Quantification of YSD enrichment across 6 campaigns. **e**, Quantification of individual binders enriched from the BC2 campaign, highlighting enriched binders selected for further validation. Blue dots, three selected binders (C1–C3); grey dots, non-enriched binders; black line, no change from parental pool. **f**, Characterization of CAR antigen binding at variable recombinant CD19 concentrations. **g**, Overview of CAR Jurkat activation screening from the BC2 campaign. **h**, Summary of CD69 activation via co-culture of C1 and C2 CARPNN-evolved binders with Ramos cell line, highlighting selected CARs. **i**, Killing curves measuring target cell growth (% intensity) over 72 h of co-culture with CAR T constructs (Ø, FMC63, C1, C2) against K562 (CD19^−^), K562–CD19^OE^ (CD19^+^) and K562–CD19^OE^–CD81^KD^ target cells at an E:T ratio of 1:1. Data are presented as mean ± s.e.m. of *n* = 3 replicate wells per condition. **j**, Schematic of occluded epitope engagement of CD19-directed minibinders from CD81 binding on target cells using the aligned FMC63–CD19–CD81 complex.

**Fig. 6 |:**
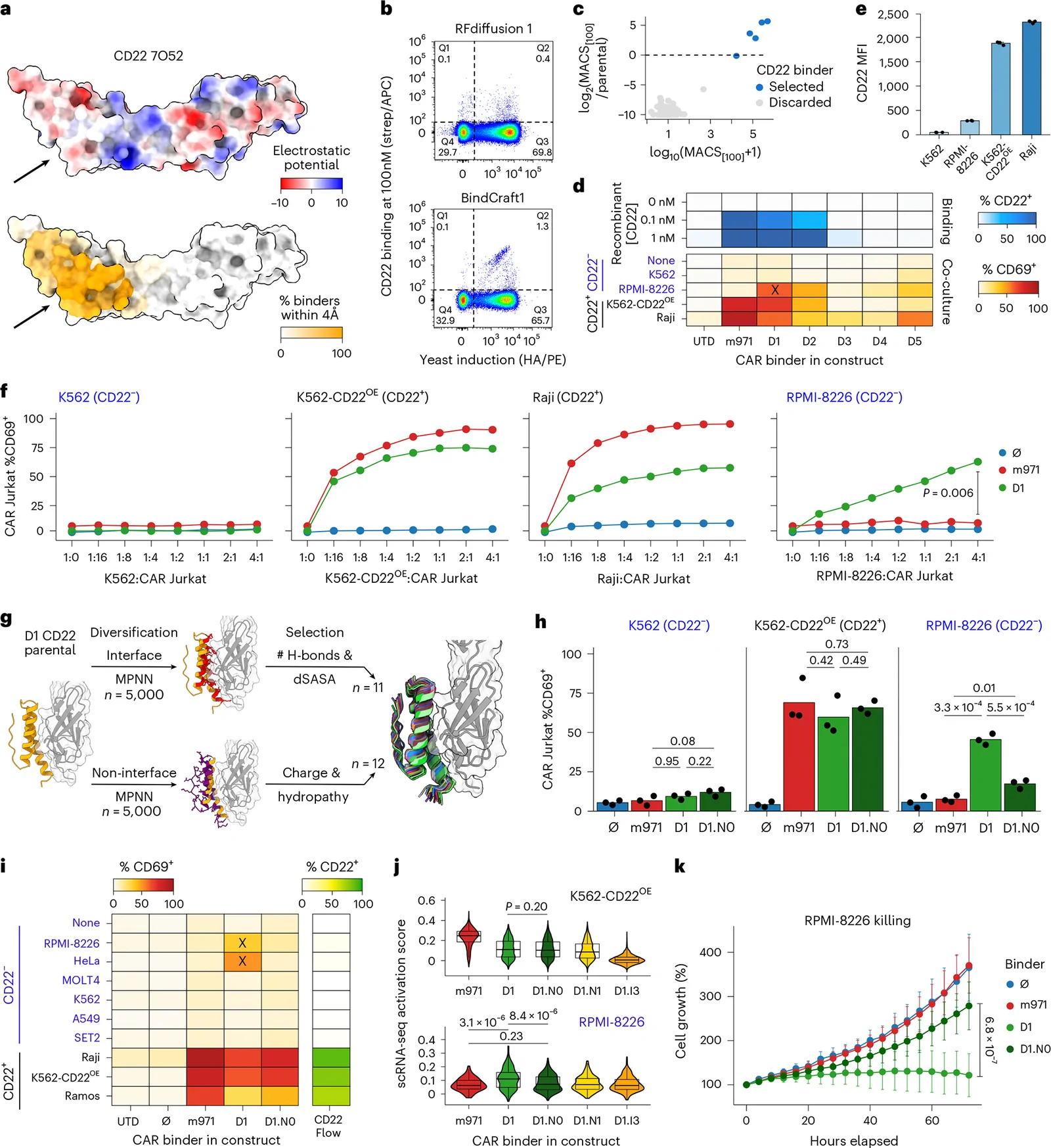
Reverting off-target binding through engineering de novo designed CD22-directed CARs. **a**, Schematic of CD22 target antigen, with the arrow highlighting the preferred binding pocket as a hydrophobic patch. Electrostatic potential is shown in kcal per mol *e* and is shown for red-blue surface colouring. **b**, Summary of YSD screening of de novo designed proteins from 2 campaigns against CD22. **c**, Identification of 4 hits from the BindCraft campaign via sequencing. **d**, CAR co-culture of 5 de novo CD22 binders (D1–D5) compared to m971 (clinical CAR). Shown is the %CD69^+^ Jurkats among GFP^+^ cells. **e**, Validation of CD22 expression of cell lines showing mean fluorescence intensity (MFI). Bars are the average of 3 biological replicates, shown individually as dots. **f**, Co-cultures of 3 CARs with variable effector-to-target (E:T) ratios. Statistical test used was Wald test of linear regression comparing D1 de novo binder to m971 clinical CAR, adjusting for E:T ratio. **g**, Diversifying CD22 binder sequences given a single binder (D1). **h**, Triplicate CAR Jurkat co-cultures with variable CAR binders. Statistical test used was two-sided Student’s *t*-test. **i**, Summary of diversified CD22 binders in CAR co-culture. ‘X’ highlights off-target activation from parental binder, D1. **j**, Activation scores from scRNA-seq of CAR binders cultured against two different cell lines (*n* = 1 donor). Statistical test used was two-sided Mann–Whitney *U*-test. Boxplots: centre line, median; box limits, first and third quartiles; whiskers, 1.5× interquartile range. **k**, Primary CAR T killing curves against RPMI- 8226 (CD22^−^) showing off-target-specific killing in the de novo D1 binder for donor triplicates. Statistical test used was Wald test of the linear regression interaction term between the D1.N0 binder and time compared to D1, adjusting for time and binder identity.

**Fig. 7 |:**
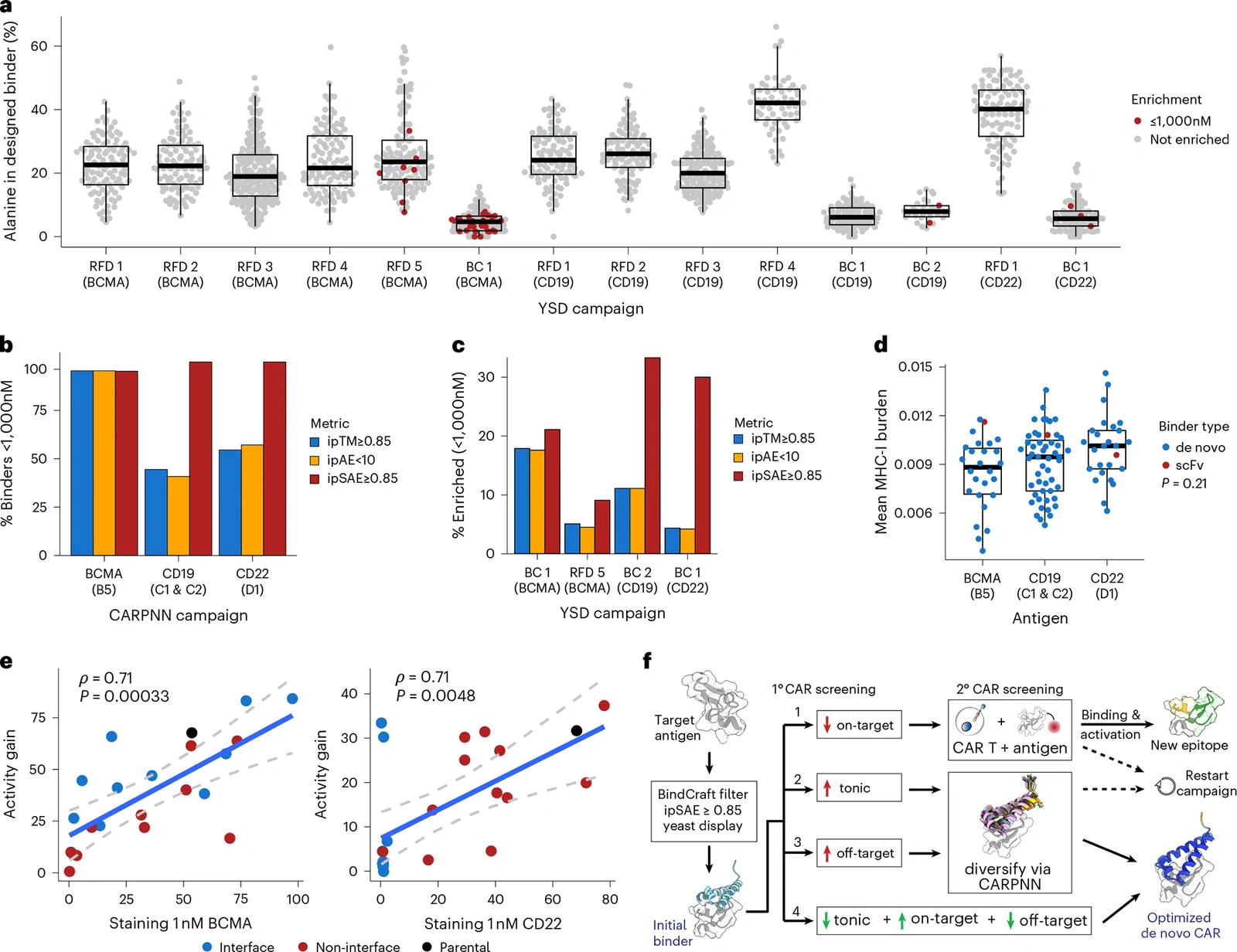
Retrospective campaign analysis reveals in silico correlates of successful CAR design. **a**, Percentage of alanine in binder sequences tested in yeast display campaigns (RFD, RFdiffusion; BC, BindCraft) (*n* = 1,589). Boxplots: centre line, median; box limits, first and third quartiles; whiskers, 1.5× interquartile range. **b**, Proportion of binders within the CARPNN workflow with binding affinity stronger than 1,000 nM using various in silico cut-offs across 3 antigens. **c**, Proportion of binders enriched in yeast surface display campaigns with binding affinity stronger than 1,000 nM using various in silico cut-offs. **d**, Comparison of the average fraction of peptides predicted to bind strongly to class-I MHC-I across all alleles supported by NetMHC (4.2)^48^ for all binders tested as CARs (*n* = 103). Statistical test used was Wald test statistic from a generalized linear model. Boxplots: centre line, median; box limits, first and third quartiles; whiskers, 1.5× interquartile range. **e**, Comparison of staining intensity via recombinant antigen at 1 nM compared to mean proportion of CD69^+^ CAR^+^ cells co-cultured with antigen-positive cells subtracted by the proportion of CD69^+^ CAR^+^ cells alone. The *x* axis denotes the proportion of binder^+^ cells bound to 1 nM of recombinant BCMA/CD22. *P* value and point estimate are reported from a two-sided Spearman correlation test. Blue line, least squares regression line; grey dotted line, 95% confidence interval. **f**, Schematic of recommended workflow for designing de novo minibinder-based CAR.

## Data Availability

Raw and processed scRNA-seq data are available at GSE310399. Source data are provided with this paper.

## Code availability

Custom code for replicating downstream analyses is available in GitHub at https://github.com/clareaulab/denovo-cart-reproducibility ref. 60. The method for diversifying and scoring CAR minibinder sequences is also available on GitHub at https://github.com/clareaulab/CARPNN ref. 61.
