# Supplementary material for: Sequence and structural determinants of efficacious de novo chimaeric antigen receptors
Source: Nat Biomed Eng. Author manuscript; Available in PMC 2026 Oct 11. (PMC13654480; doi:10.1038/s41551-026-01790-9)
Supplement: Supplementary file 1 [file NIHMS2212395-supplement-Supplementary_Information.pdf]

nature biomedical engineering

Supplementary information https://doi.org/10.1038/s41551-026-01790-9

Sequence and structural determinants of efficacious de novo chimaeric antigen receptors

In the format provided by the authors and unedited

Supplemental Information

Table of contents

Supplemental Note 1. Details concerning parental backbone generation
Supplemental Note 2. Preparation of yeast libraries for sequencing
Supplemental Note 3. Generation of engineered cell lines
Supplemental Note 4. Single-cell RNA-seq analyses
Supplemental Note 5. Additional *de novo* binder campaigns against CD19
Supplemental Note 6. CD22 *in silico* off-target screen
Supplemental Note 7. In-depth characterization of *in silico* metrics

Supplementary Figures
Supplementary Fig 1. *De novo* binder campaigns targeting the FMC63 epitope of CD19
Supplementary Fig 2. *In silico* correlates of minibinder experimental success

References

Supplemental Note 1. Details concerning parental backbone generation

RFdiffusion

To generate *de novo* binders against tumor-associated antigens, we first used RFdiffusion to generate protein binder backbones against the crystal structure of the extracellular domain of BCMA bound to its native ligand APRIL (PDB: 1XU2). We launched three campaigns targeting two sets of hotspots consisting of hydrophobic residues: one campaign targeting the outward-facing leucines in positions 17, 26, and 35 (BCMA RFdiffusion Campaign 1), and another two sets of campaigns targeting hydrophobic residues consisting of the phenylalanine at position 14, leucine at position 18, and alanine at position 21 (BCMA RFdiffusion Campaign 2, BCMA RFdiffusion Campaign 3). In all campaigns, we generated protein backbones with lengths ranging from 30-90 without conditioning on structural scaffolds. Backbones with no alpha-carbon less than 10Å away from BCMA were also discarded.

After failing to yield high-affinity binders in our initial campaigns, we sought to target new sets of epitopes that may be more conducive to protein-protein interaction. Using PeSTo[1], a deep-learning model that predicts binding surfaces on biomolecules, we identified three residues predicted to have a high likelihood of forming protein-protein interactions on BCMA (1XU2, Chain R), leucine17 (score = 0.95), histidine18 (score = 0.9), and alanine19 (score = 0.88) (BCMA RFdiffusion Campaign 4).

For all RFdiffusion campaigns, we used the filtered backbones as input into ProteinMPNN[2] with recommended settings[3] and generated 10 putative sequences assigned per backbone. To identify predicted high-confidence binders, we filtered via the "AF2 initial guess" protocol, which enables fast screening of binders. In brief, the putative binder sequences are refolded against the target structure, and high-quality interactions were filtered such that the resulting co-fold had a complex pLDDT > 80 and pAE interaction < 10. Binders passing *in silico* metrics were then sorted by ascending pAE interaction, and the top 100 sequences with the lowest pAE interaction per backbone were selected for screening via yeast display.

For CD19, we applied the same RFdiffusion-ProteinMPNN-AF2 pipeline to generate 100 protein binders against the crystal structure of the extracellular domain of CD19 bound to FMC63 (PDB: 7URV), with hotspots selected to be isoleucine at position 73, valine at position 241, and leucine at position 247 (CD19 RFdiffusion Campaign 1). Notably, the *in silico* success rate of backbone generation against CD19 was much lower than that of BCMA, likely in part due to the much larger target structures. Next, we used a similar PeSTo-based approach to identify four residues on CD19 (PDB 7URV, chain C) with high probability for protein interaction: leucine188 (score = 0.81), methionine190 (score = 0.85), threonine195 (score = 0.89), and tryptophan197 (score = 0.88) (CD19 RFdiffusion Campaign 2). These residues are then provided as hotspots to RFdiffusion, and the same selection strategy was applied to generate 100 CD19 binders for experimental validation.

After the second round of BCMA and CD19 campaigns failed to yield high-affinity binders, we hypothesized that the lack of binding was not due to insufficient contact with the target but rather the lack of stable binder structures. To test this hypothesis, we removed the backbone diversity filter and decided to constrain the overall fold of the binders to previously validated protein scaffolds[4] as provided by the RFdiffusion developers and used the fold-conditioning protocol to generate 134 binders against BCMA (BCMA RFdiffusion Campaign

5) and 196 binders against CD19 (CD19 RFdiffusion Campaign 3, CD19 RFdiffusion Campaign 4) with the same epitope specification and selection strategy as previous campaigns. This strategy generated a moderate affinity BCMA binder, while no CD19 binder was obtained.

For CD22, a similar strategy was used. Using the crystal structure of the Ig domains 1-3 of CD22 (PDB: 5VKJ, chain A[5]) as input, we did not identify any residues with high PeSTo scores. Thus, we selected three hydrophobic residues in close proximity as hotspot inputs: Leucine 161, Leucine 193, and Phenylalanine 206. A fold-conditioned RFdiffusion campaign was launched with the same selection as the previous campaign to generate 102 CD22 binders (CD22 RFdiffusion Campaign 1).

BindCraft

Motivated by the success of BindCraft in generating binders against EGFR in a public competition[6], we launched *in silico* campaigns against BCMA, CD19, and CD22 using BindCraft, providing the same antigen crystal structures provided to RFdiffusion as input. We observed high-confidence binder structures against both antigens without pre-specifying hotspot residues, and then generated the remaining binders using similar settings. In the BCMA BindCraft campaign (BCMA BindCraft Campaign 1), binders of length 60-65 were specified. The default four-stage multimer setting was used to generate hallucination trajectories; the binders are then subject to the default filters; and the campaign concluded after ~300 filter-passing designs were reached. The filter-passing BCMA binders are then ranked by ipTM as recommended, and after deduplicating sequences derived from the same protein backbone, the top 125 sequences were selected for experimental testing.

In the first CD19 BindCraft campaign (CD19 BindCraft Campaign 1), the binders were similarly ranged between 50-65 amino acids, and the default hallucination trajectory settings were used. However, we noticed a low initial generation success rate and experimented with other setting configurations, varying parameters such as removing the target template sequence during design and re-prediction (the “default_4stage_multimer_flexible” setting), providing a binder template during re-prediction (the “default_4stage_multimer_hardtarget” setting), and increasing the helicity weight penalty (the “betasheet_4stage_multimer_hardtarget” setting). The design binders passing default filters using different settings were then aggregated, and the top 100 binders with the highest ipTM were selected.

For the second CD19 BindCraft campaign (CD19 BindCraft Campaign 2), we reasoned that the lack of binding was due to insufficient contact surface between the de novo binder and CD19. As such, we adjusted the generation settings to binders between length 100-200 and used similar filters and diverse trajectory settings as the first CD19 BindCraft campaign. For this campaign, we decided to implement a more stringent pLDDT filter of 0.9 and proceeded with 30 binders with the highest pLDDT. The lower selected binder number was to accommodate synthetic DNA orders as eBlocks rather than o-Pools to minimize costs.

To design binders against CD22, we reasoned that targeting an epitope that is more membrane proximal may increase CAR efficacy[7]. As such, we decided to use the crystal structure of the d6-d7 domain of CD22 bound to the pre-clinical anti-CD22 scFV m971 (PDB: 7O52). Given our success in generating BCMA and CD19 binders using BindCraft, we used similar hallucination trajectory settings and filters to generate ~500 *in silico* binders between lengths 50- 65 and picked the top 100 binders ranked by ipTM (CD22 BindCraft Campaign 1).

Supplemental Note 2. Preparation of yeast libraries for sequencing

Plasmid DNA was isolated from yeast using a modified QIAGEN Plasmid Midi Kit (Qiagen, Cat. 12143) protocol. Briefly, 2g of yeast cells were harvested and converted to spheroplasts in SCE buffer (1M sorbitol, 0.1 M sodium acetate, 60mM EDTA, pH 7.0) with zymolyase (5 mg) and β-mercaptoethanol (30 µL) for 1 h at 37 °C. Spheroplasts were then lysed, purified, and the plasmid was eluted following the manufacturer's protocol. Enriched plasmid libraries were modified by PCR amplification to append sequencing-compatible adapter elements with a forward primer pool 5'-TCGTCGGCAGCGTCAGATGTGTATAAGAGACAG$(N)_{0\text{-}2}$GGTCGGCTAGCCATATG-3' and reverse primer pool 5'-GTCTCGTGGGCTCGGAGATGTGTATAAGAGACAG$(N)_{0\text{-}2}$CTTTTGTTCGGATCC-3'. PCR amplicons were purified using SPRIselect DNA size bead cleanup (Beckman Coulter, B23318), and Illumina i5 and i7 index sequences were subsequently appended to generate sequencing-ready libraries. Final libraries were quantified and size-verified by TapeStation (Agilent) prior to dilution and loading onto the Element Aviti sequencer. Library abundance was determined by building a custom index of DNA sequences using kallisto[8], and each library was quantified using default parameters for a paired-end sequencing library.

Supplemental Note 3. Generation of engineered cell lines

Overexpression cell lines

To generate antigen-overexpressing cell lines, lentiviral particles were produced by co-transfecting HEK293T cells with the LentiV_Blast vector (Addgene, 111887) encoding the gene of interest (BCMA, CD22, CD19), the packaging plasmid psPAX2, and the envelope plasmid VSVG using FuGENE HD transfection reagent (Promega, Cat. E2311). Briefly, 6×10^6 HEK293T cells were seeded in 10 cm dishes containing 10 mL DMEM supplemented with 10% FBS and 1% penicillin–streptomycin 24 hours before transfection. On the day of transfection, 6 µg of transfer vector, 3 µg of psPAX2, and 1 µg of VSV-G were combined in 500 µL Opti-MEM with 18 µL FuGENE HD, incubated at room temperature for 15 minutes, and added dropwise to the HEK293T cells. At 18 hours post-transfection, the medium was replaced with 10 mL of fresh DMEM. Lentiviral supernatants were collected at 48 and 72 hours post-transfection, pooled, and filtered through a 0.45 µm syringe filter (Fisher Scientific, 09-754-21). Filtered supernatants were concentrated using Lenti-X Concentrator (Takara Bio, Cat. 631232) according to the manufacturer's protocol to a final volume of 2 mL and titered before transduction. K562 target cells were transduced at the optimal titer determined and selected with puromycin (2.5 µg/mL) for ~ 1.5 weeks to generate stable cell lines.

Knockdown cell lines

CD81 knockdown K562 and Raji cell lines were generated using the PLKO.1 lentiviral shRNA system. Lentiviral particles encoding CD81-targeting shRNAs
F- CCGGCCTGCTCTTCGTCTTCAATTTCTCGAGAAATTGAAGACGAAGAGCAGGTTTTTG,
R- AATTCAAAAACCTGCTCTTCGTCTTCAATTTCTCGAGAAATTGAAGACGAAGAGCAGG;
TRC609) or a non-targeting shRNA scramble were produced following the same protocol outlined in the overexpression method. Supernatants were collected, filtered, concentrated, and titered identically. Target cells were transduced and selected with puromycin (2.5 µg/mL) for 1.5 weeks to generate stable cell lines. Successful knockdown of CD81 expression was confirmed via flow cytometry.

mCherry-expressing cell lines

To enable fluorescence-based killing readouts (Incucyte), antigen-overexpressing lines (BCMA, CD22, and CD19) were generated following the same lentiviral production, transduction, and puromycin selection workflow described above. Successful double expression of antigen and mCherry-tag was confirmed by flow cytometry before use.

Supplemental Note 4. Single-cell RNA-seq analyses

CAR T cells from a single donor were co-cultured with target antigen–positive or target antigen-negative cells for 24 h, harvested, and enriched by FACS for $CD3^+$ T cells. Cells were washed in cell Staining Buffer (BioLegend, Cat. 420201), blocked with TruStain FcX (BioLegend, Cat. 422301) for 10 min at 4 °C, and stained with hashtag oligonucleotide (HTO) antibodies (0.25 µg per sample; TotalSeq-C, as appropriate) for 30 min at 4 °C. After two washes, individual CAR T populations were counted, assessed for viability, and combined at the desired ratios for a minimum of 30k total cells.

Single cells were partitioned into GEMs (gel bead-in-emulsions) on a Chromium Controller (10x Genomics; Single Cell 5′ kit, Cat. PN-1000695) following the manufacturer's protocol with Feature Barcoding for hash tag oligos (HTOs; 10x Genomics, Cat. 1000703). Reverse transcription was performed in-GEM, followed by emulsion breakage, cDNA cleanup, and amplification. Separate gene-expression and HTO libraries were constructed per 10x recommendations. Library quality and size distributions were verified by TapeStation (Agilent) and concentration by Qubit (Thermo). Pooled libraries were sequenced on an Element AVITI instrument using paired-end chemistry with 10x Genomics compatible read lengths. Different CARs were multiplexed with 5'-compatible Totalseq-C reagents. Per-cell hash quantifications were estimated using kallisto[8] for the relevant hashing antibodies, and per-cell hash assignments were annotated using default Seurat settings[9].

After demultiplexing and hashing assignment, each sample's raw count matrix was loaded as Seurat objects[10] for downstream analysis. Filtering based on total RNA count, unique RNA detected, and proportion of mitochondrial reads was performed to remove low-quality cells. For each sample, read counts were normalized and scaled, and dimensionality reduction was performed to identify cell neighborhoods, followed by Leiden clustering. Cells in clusters lacking *CD3E* expression were considered contaminants and were removed, after which the filtered sets of cells were identically reprocessed in Seurat. To account for cell cycle effects, cell cycle scoring was performed, and Harmony[11] was applied to produce a phase-regressed embedding. To assign $CD4^+$ and $CD8^+$ subtypes, cells were unsupervised clustered by expression profile, and the average CD4 expression and the average sum of *CD8A* and *CD8B* expression were computed per cluster. Cells in clusters with higher average CD4 expression were then assigned $CD4^+$ and vice versa. Differentially expressed genes were identified using a default implementation of the Wilcoxon rank sum test between different binders with p-values adjusted for multiple hypothesis testing via the Benjamini-Hochberg procedure. Genes overexpressed by cells expressing the evolved BCMA binder B5.I0 compared to cells expressing the parental BCMA binder B5 were then filtered using a log2 fold-change threshold above 0.25 and adjusted p-value below 0.05 to obtain the minibinder activation gene set. The activation module scores for CD22 experiments were then calculated using this gene set via the AddModuleScore function from Seurat.

Supplemental Note 5. Additional *de novo* campaigns against CD19

To target CD19 while avoiding epitope occlusion by CD81, we reasoned that targeting the FMC63 epitope (residues 136-145, 197-205) with an alternative binder format or using newer tools with higher epitope-conditioned binder sampling efficiency could improve our success rate. To accomplish this, we launched a new campaign via BoltzGen[12]. This protein diffusion model has been successfully applied in our group and others in the community[13] to generate *de novo* binders against multiple targets. In particular, BoltzGen could design *de novo* variable domains of heavy-chain-only (VHHs), which can contact the target antigen via flexible Complementarity-Determining Region (CDR) loops similar to how FMC63 contacts CD19. We reasoned this would be an efficacious approach to reducing the hydrophobicity bias that we recurrently observed with tools like BindCraft[14]. In parallel, we also launched a campaign targeting the FMC63 epitope with minibinders using RFdiffusion3[15], which is reported to improve *in silico* binder sampling efficiency compared to the original RFdiffusion[16].

Using RFdiffusion3 and BoltzGen, we generated binders with high confidence metrics that were predicted to target the FMC63 epitope, distal from the CD81 binding pocket **(Methods, Supplementary Fig. 1a,b).** In the BoltzGen campaign, 67 VHH designs with either high default BoltzGen ranking or high ipSAE were selected for experimental testing **(Supplementary Fig. 1c)**. The sequences were then synthesized and cloned into the pYAL yeast display backbone with a C-terminal HA tag downstream of the VHH so that the CDR-rich N-terminal face remains exposed for antigen engagement. Unfortunately, no binding was detected upon yeast transformation and antigen staining up to 100nM concentration **(Supplementary Fig. 1d)**. In the RFdiffusion3 campaign, 102 binders with ipSAE > 0.6 were selected for experimental testing **(Supplementary Fig. 1e).** We again used the pETCON(-) backbone as in our previous minibinder campaigns **(Methods)**. Again, no binding was detected when the binders were transformed into yeast, induced, and incubated with up to 100 nM of recombinant CD19 **(Supplementary Fig. 1f).**

The lack of experimentally confirmed binders in either campaign, despite dozens of binders satisfying *in silico* metrics, suggests that the FMC63 epitope on CD19 may represent a particularly challenging design target due to its intrinsic biophysical properties that are not adequately captured by current design and prediction models. As many immunologically relevant binding sites contain predominantly polar residues (including FMC63) or are otherwise biophysically demanding, future efforts to develop computational binder design methods will require sampling potential protein binders from a broader energy landscape. If achieved, such methods could improve protein-antibody interaction prediction and enable targeting of “cold-spot” residues that the current generation of methods cannot adequately achieve.

Methods

BoltzGen VHH Campaign against FMC63 Epitope of CD19

A total of 14,236 *de novo* VHHs targeting the FMC63 epitope of CD19 were generated via BoltzGen[17] using the default parameters for VHH under the “nanobody-anything” protocol. Among binders passing default filters (*n*=667), designs were manually inspected, and those predicted to bind within the CD81-overlapping epitope were excluded. The top 39 candidates were then selected based on the default BoltzGen nanobody ranking criteria. In parallel, the top 500 BoltzGen-ranked designs were refolded by Boltz2 and rescored using ipSAE, from which an

additional 28 candidates with the highest ipSAE were selected. In total, 67 nanobody designs were synthesized via IDT eBlocks and cloned into the pYAL yeast display backbone with a C-terminal HA tag downstream of the binder for surface expression detection. Our yeast surface display (YSD) protocol proceeded using the same induction and flow-based readouts as the other campaigns in this manuscript.

RFdiffusion3 Minibinder Campaign against FMC63 Epitope of CD19

Similar to our prior RFdiffusion campaigns, we generated 1,000 backbones targeting the FMC63 epitopes and assigned 10 sequences per backbone via SolubleMPNN[18]. These generated sequences were then co-predicted with the CD19 sequence via Boltz2. A BindCraft-adapted filter based on Boltz2 metrics was then applied to filter for binders. In addition, a binder must satisfy all of the following conditions: single-sequence mode monomer lDDT > 80, monomer RMSD < 2.5, ipSAE > 0.6, net charge < 0, and must have more than 5 residues within 4Å of the FMC63 epitope. In total, 102 binders passed these filters and were selected for experimental testing.

Supplemental Note 6. CD22 *in silico* off-target screen

To identify the off-target interactions we observed from the initial CD22 D1 binder, we reasoned that the off-target interaction may be mediated by the binder binding to surface proteins that are sufficiently expressed in $CD22^-$ cell lines where the CAR is activated but are not sufficiently expressed in $CD22^-$ cell lines where the CAR is not activated. Using the curated membrane protein list from the Human Protein Atlas (*n*=2,632) and the cell line expression profiles from DepMap, we identified 19 candidates that are positive for RPMI-8226 and HeLa but are negative for K562, SET2, A549 using either 100 or 1,000 TPM as the cutoff. These 19 candidate off-target proteins were then folded against D1 and the rescued binder D1.N0 via Boltz. Among all predicted interactions, only CXCR4 had significantly higher ipSAE in D1 compared to the evolved D1.N0. However, upon inspection of the predicted structure, in all 5 diffusion samples of the predicted D1-CXCR4 complex, the interaction was occurring through the cytosolic end of CXCR4 that is inaccessible to the cell surface (PDB: 8U4R), which suggests the predicted interaction is likely spurious.

Supplemental Note 7. In-depth characterization of *in silico* metrics

To characterize the performance of various *in silico* metrics for distinguishing binders at different affinity thresholds, we further analyzed yeast surface display across all targets at variable concentrations (100nM and 1000nM). Overall, the ranking of the most predictive features remained relatively robust across different affinity cutoffs, with average ipSAE max having the highest overall AUROC in either affinity cutoff (**Supplementary Fig. 2a,b)**. However, the ranking can vary when broken down by antigen, where complex pLDDT was more predictive of binders with yeast surface display enrichment in CD19 compared to ipSAE max (**Supplementary Fig. 2c,d)**.

Given the relative strong performance of ipSAE at predicting yeast surface display enriched binders, and the growing interests of the field at applying ipSAE to identify binders[13,19,20], we investigated the relationship between ipSAE variants (hereby referred to as ipSAE max, ipSAE min, ipSAE binder->target, ipSAE target->binder respectively) and their relative performances in separating enriched binders in yeast surface display (**Supplementary Fig. 2e**) and their correlation against estimated on-cell Kd (**Supplementary Fig. 2f**). We found that in either setting, ipSAE max had clearer separation of enriched binders at fixed threshold than ipSAE min and that Chn1:target->Chn2:binder was mostly correlating with ipSAE max (**Supplementary Fig. 2g**). Together, these results support the use of ipSAE max as a robust general-purpose metric for binder prioritization and highlights the optimal scoring metric may vary by antigen.

SUPPLEMENTAL FIGURES

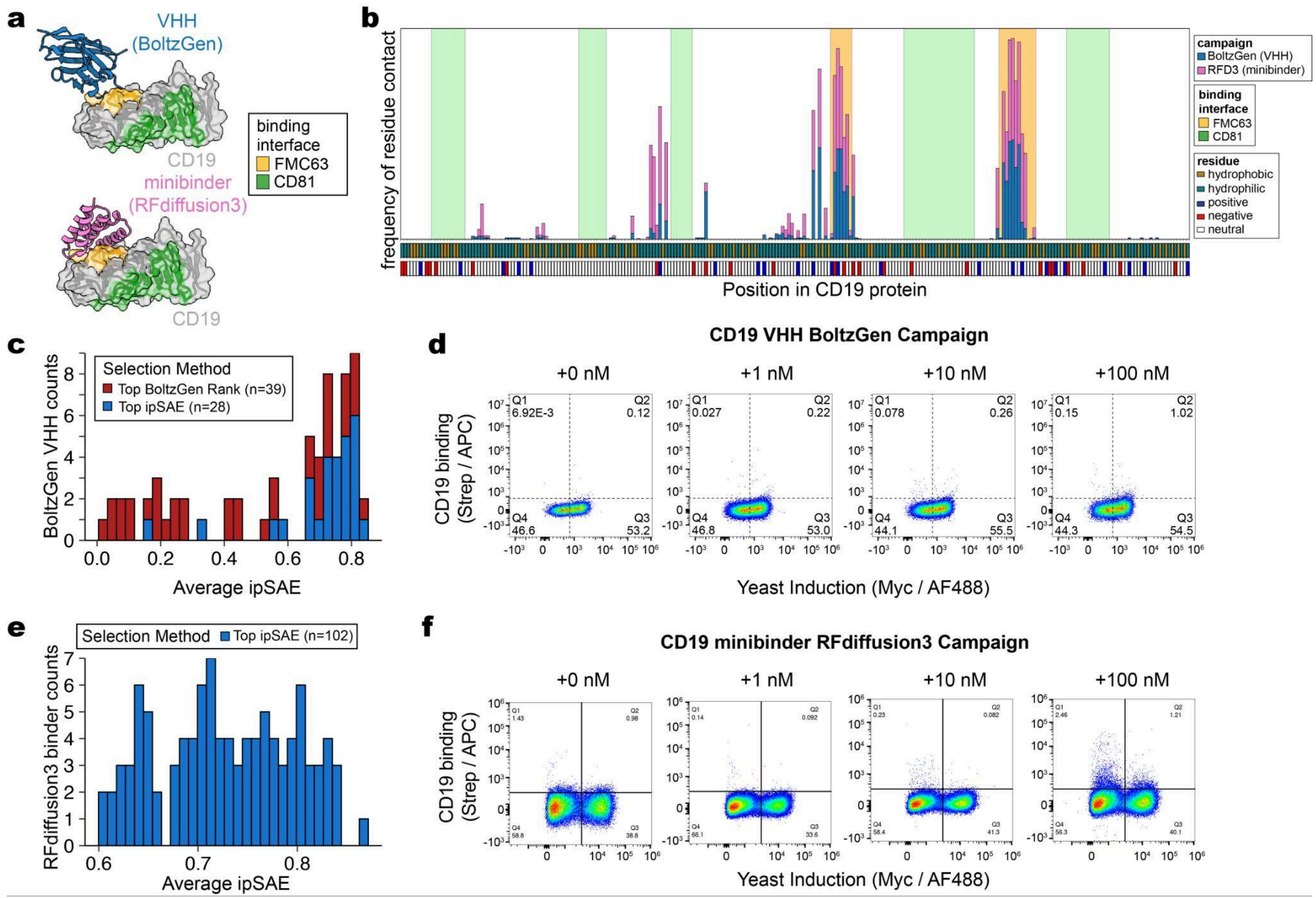

Supplementary Fig. 1. *De novo* binder campaigns targeting the FMC63 epitope of CD19. a, Predicted structures of representative binders in complex with CD19 in the BoltzGen VHH campaign and the RFdiffusion3 campaign. **b,** Residue contact frequency of the binders in each campaign with respect to CD19 amino acid positions. **c,** Average ipSAE of BoltzGen VHH selected for experimental screening. **D,** Yeast surface display result of the CD19 BoltzGen VHH campaign. **e,** Average ipSAE of RFdiffusion3 binders selected for experimental screening. **F,** Yeast surface display result of the CD19 RFdiffusion3 minibinders campaign.

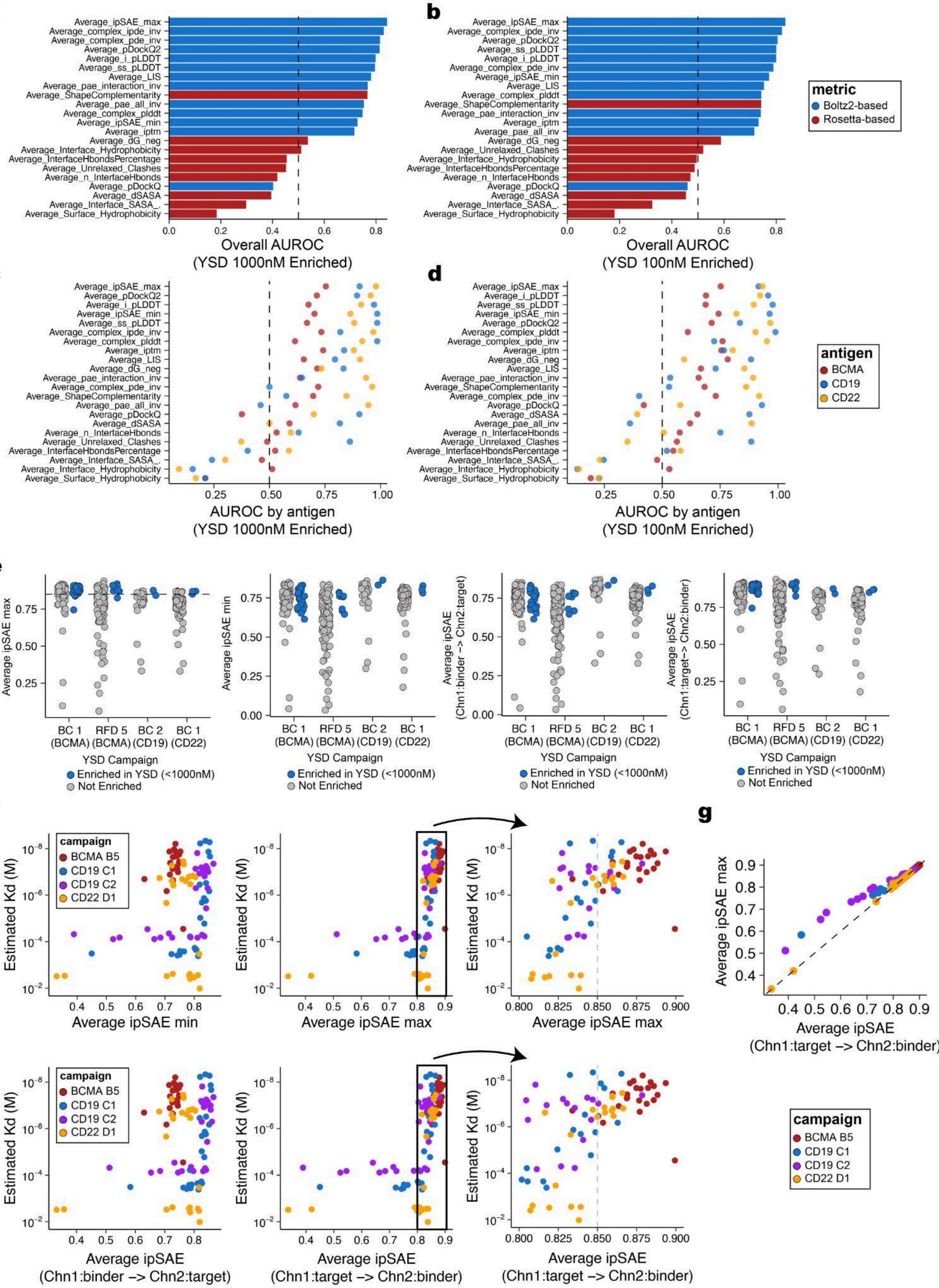
a
Average_ipSAE_max
Average_complex_ipde_inv
Average_complex_pde_inv
Average_pDockQ2
Average_i_pLDDT
Average_ss_pLDDT
Average_LIS
Average_pae_interaction_inv
Average_ShapeComplementarity
Average_pae_all_inv
Average_complex_plddt
Average_ipSAE_min
Average_iptm
Average_dG_neg
Average_Interface_Hydrophobicity
Average_InterfaceHbondsPercentage
Average_Unrelaxed_Clashes
Average_n_InterfaceHbonds
Average_pDockQ
Average_dSASA
Average_Interface_SASA_.
Average_Surface_Hydrophobicity
Overall AUROC
(YSD 1000nM Enriched)
b
Overall AUROC
(YSD 100nM Enriched)
metric
Boltz2-based
Rosetta-based
c
AUROC by antigen
(YSD 1000nM Enriched)
d
AUROC by antigen
(YSD 100nM Enriched)
antigen
BCMA
CD19
CD22
e
Average ipSAE max
Average ipSAE min
Average ipSAE (Chn1:binder -> Chn2:target)
Average ipSAE (Chn1:target-> Chn2:binder)
BC 1 (BCMA)
RFD 5 (BCMA)
BC 2 (CD19)
BC 1 (CD22)
YSD Campaign
Enriched in YSD (<1000nM)
Not Enriched
f
campaign
BCMA B5
CD19 C1
CD19 C2
CD22 D1
Estimated Kd (M)
Average ipSAE min
Average ipSAE max
Average ipSAE (Chn1:binder -> Chn2:target)
Average ipSAE (Chn1:target -> Chn2:binder)
g
Average ipSAE max
Average ipSAE (Chn1:target -> Chn2:binder)
campaign
BCMA B5
CD19 C1
CD19 C2
CD22 D1

Supplementary Fig. 2*. In silico* correlates of minibinder experimental success. a-b, Area under the receiver operating curve (AUROC) of each in silico metric across all RFdiffusion and BindCraft campaigns, with binders defined as yeast surface display enrichment at **a,** 1000nM or **b,** 100nM. **c-d,** AUROC of each in silico metric across antigens with binders defined as yeast surface display enrichment at **c,** 1000nM or **d,** 100nM. **e,** Performance of ipSAE variants in campaigns with at least one experimentally validated minibinder. **f,** Relationship between ipSAE variants and estimated Kd in MPNN campaigns. **g,** Relation between ipSAE variants.

REFERENCES

1. Krapp, L. F., Abriata, L. A., Cortés Rodriguez, F. & Dal Peraro, M. PeSTo: parameter-free geometric deep learning for accurate prediction of protein binding interfaces. *Nat. Commun.* **14**, 2175 (2023).
2. Dauparas, J. *et al.* Robust deep learning-based protein sequence design using ProteinMPNN. *Science* **378**, 49–56 (2022).
3. Bennett, N. R. *et al.* Improving de novo protein binder design with deep learning. *Nat. Commun.* **14**, 2625 (2023).
4. Watson, J. L. *et al.* De novo design of protein structure and function with RFdiffusion. *Nature* **620**, 1089–1100 (2023).
5. Ereño-Orbea, J. *et al.* Molecular basis of human CD22 function and therapeutic targeting. *Nat. Commun.* **8**, 764 (2017).
6. Cotet, T.-S. *et al.* Crowdsourced protein design: Lessons from the Adaptyv EGFR binder competition. *bioRxiv* 2025.04.17.648362 (2025) doi:10.1101/2025.04.17.648362.
7. Ereño-Orbea, J. *et al.* Structural details of monoclonal antibody m971 recognition of the membrane-proximal domain of CD22. *J. Biol. Chem.* **297**, 100966 (2021).
8. Bray, N. L., Pimentel, H., Melsted, P. & Pachter, L. Near-optimal probabilistic RNA-seq quantification. *Nat. Biotechnol.* **34**, 525–527 (2016).
9. Stoeckius, M. *et al.* Cell Hashing with barcoded antibodies enables multiplexing and doublet detection for single cell genomics. *Genome Biol.* **19**, 224 (2018).
10. Hao, Y. *et al.* Integrated analysis of multimodal single-cell data. *Cell* **184**, 3573-3587.e29 (2021).
11. Korsunsky, I. *et al.* Fast, sensitive and accurate integration of single-cell data with Harmony. *Nat. Methods* **16**, 1289–1296 (2019).
12. Stark, H. *et al.* BoltzGen: Toward universal binder design. *bioRxivorg* 2025.11.20.689494 (2025) doi:10.1101/2025.11.20.689494.

13. Nipah Competition Results. *Proteinbase* https://proteinbase.com/collections/nipah-binder-competition-results.

14. Pacesa, M. *et al.* One-shot design of functional protein binders with BindCraft. *Nature* **646**, 483–492 (2025).

15. Butcher, J. *et al.* De novo design of all-atom biomolecular interactions with RFdiffusion3. *bioRxivorg* 2025.09.18.676967 (2025) doi:10.1101/2025.09.18.676967.

16. Watson, J. L. *et al.* De novo design of protein structure and function with RFdiffusion. *Nature* **620**, 1089–1100 (2023).

17. Wohlwend, J. *et al.* Boltz-1 democratizing biomolecular interaction modeling. *bioRxivorg* 2024.11.19.624167 (2025) doi:10.1101/2024.11.19.624167.

18. Goverde, C. A. *et al.* Computational design of soluble and functional membrane protein analogues. *Nature* **631**, 449–458 (2024).

19. Overath, M. D. *et al.* Predicting experimental success in DE Novo binder design: A meta-analysis of 3,766 experimentally characterised binders. *bioRxiv* 2025.08.14.670059 (2025) doi:10.1101/2025.08.14.670059.

20. Didi, K. *et al.* Latent Generative Search unlocks de novo Design of Untapped Biomolecular Interactions at Scale. https://research.nvidia.com/labs/genair/proteina-complexa/assets/proteina_complexa_validation.pdf (2026).
